# Discovering the Next-Generation Plant Protection Products: A Proof-of-Concept via the Isolation and Bioactivity Assessment of the Olive Tree Endophyte *Bacillus* sp. PTA13 Lipopeptides

**DOI:** 10.3390/metabo11120833

**Published:** 2021-12-02

**Authors:** Evgenia-Anna Papadopoulou, Apostolis Angelis, Lemonia Antoniadi, Konstantinos A. Aliferis, Alexios-Leandros Skaltsounis

**Affiliations:** 1 Laboratory of Pesticide Science, Department of Crop Science, Agricultural University of Athens, 11855 Athens, Greece; evina.papadopoulou@aua.gr; 2 Department of Pharmacognosy and Natural Products Chemistry, Faculty of Pharmacy, University of Athens, 15771 Athens, Greece; aangjel@pharm.uoa.gr (A.A.); monikaant@pharm.uoa.gr (L.A.); 3 Department of Plant Science, McGill University, Macdonald Campus, Ste-Anne-de-Bellevue, QC H9X 3V9, Canada

**Keywords:** countercurrent partition chromatography, endophytic microorganisms, fengycins, iturins, natural products, olive anthracnose, plant protection, surfactins

## Abstract

Endophytic microorganisms (EMs) have recently attracted interest for applications in plant protection, mainly due to their bioactive compound-producing capacity. Therefore, we underwent the task of isolating olive tree EMs and investigating their bioactivity against the devastating pathogen *Colletotrichum acutatum*. Several EMs were isolated; however, the *Bacillus* sp. PTA13 isolate exhibited the highest toxicity to the phytopathogen. Bacteria of the genus *Bacillus* exhibit superior bioactive metabolite-producing capacity, with the lipopeptides (LPs) of surfactin, iturin, and fengycin groups being the most studied. A total LP extract and several fractions were obtained, and their bioactivity was assessed against *C. acutatum* strains. LPs of the major surfactin, iturin, and fengycin groups and the minor gageotetrin and bacilotetrin groups were annotated. The results confirmed the bioactivity of the major LPs, with fengycins being the most fungitoxic. Interestingly, the minor LP fraction exhibited selective toxicity to the fungicide-resistant *C. acutatum* isolate, an observation that highlights the significance of our approach to comprehensively mine the total LP extract. This work represents a proof of concept of the exploitation of EMs in customized olive tree plant protection and aligns well with strategies that focus on the sustainability and safety of food production via the development of next-generation plant protection products.

## 1. Introduction

The quest for novel and/or alternative sources of bioactivity is necessary to address the challenges faced by the agrochemical sector in securing large quantities of high-quality food for the exponentially growing human population. Nonetheless, there is no doubt that conventional plant protection products (PPPs) represent the backbone of the agri-food sector [1,2], with more than four million tones being released annually into the agroecosystems worldwide [3]. Such dependency on conventional PPPs represents a great shortcoming for the sector, considering the inability to discover and develop bioactive molecules that exhibit new mode(s) of action (MoA) as PPPs [4,5]. The aforementioned make the exploitation of new sources of bioactivity and the development of novel plant protection strategies a necessity.

During the past few years, endophytic microorganisms (EMs) have become the focus of the research on natural product discovery. This is mainly attributed to the fact that they produce a vast array of compounds with unique structures and physicochemical properties that exhibit variable bioactivities [6,7]. Additionally, recent evidence suggests a role of EMs in the regulation of plant metabolism [8], nutrient uptake [9], and responses to biotic and/or abiotic stresses [10,11]. The symbiotic relationship that EMs have developed through the long evolutionary process with their counterparts appears to be under the control of a complex regulatory mechanism [12].

Within this context, it seems that EMs represent an excellent and alternative source of bioactivity with great potential for applications in plant protection [13,14]. However, such potential remains largely unexploited, and the knowledge of the underlying operating mechanisms is fragmented. EMs could be exploited either per se as biological PPPs or their metabolites could be used as PPPs of natural origin. Nevertheless, the isolation of natural products in their pure form is a laborious and demanding task [15,16].

To date, several endophytic bacterial and fungal species have been isolated from non-infected plant tissues [17,18,19]. Focusing on the former, species of the genus *Bacillus* are among the most studied ones, mainly due to their endospore-forming capacity, which is a feature important for their formulation and stability [20,21], and their superior capacity to synthesize a vast array of bioactive compounds [22]. An important chemical group of such compounds is that of lipopeptides (LPs), with surfactins, iturins, and fengycins being its major subgroups [23,24,25,26]. They exhibit variable bioactivity, which is mainly attributed to their amphiphilic structure (polar peptide ring linked to a hydrophobic fatty acid chain), with their antifungal, antibiotic, and anticancer activities being thoroughly studied [27,28,29,30,31]. Furthermore, there is evidence on their capacity to trigger plant defense mechanisms [32,33].

Based on the aforementioned, as a proof of concept, EMs were isolated from tissues of olive trees (*Olea europaea* L. cv. Koroneiki), and the bioactivity of the microorganisms per se or their bioactive metabolites were assessed against isolates of the olive tree pathogenic fungus *Colletotrichum acutatum* species complex [34]. The pathogen causes olive anthracnose, one of the most devastating diseases of olive trees, resulting in heavy qualitative and quantitative yield losses. The plant protection of olive tree was the main focus of the research, since its cultivation is a cornerstone for the local economies within Mediterranean countries, with Spain, Italy, Greece, and Portugal accounting for the 55, 23, 15, and 7%, respectively, of the EU total area of olive trees [35].

The developed pipeline for the discovery and development of a new source of bioactivity using an EM of the crop in focus, represents a “customized” plant protection approach (e.g., combating pests and pathogens of plant species by using a host-specific EM), which, to the best of our knowledge, is original and innovative. Furthermore, the remit of this research is in alignment with the vision of the EU as outlined in the “European Green Deal” guide on the sustainability and safety of food production, which focuses on the development of the next-generation PPPs.

## 2. Results and Discussion

### 2.1. Olive Tree Endophytic Microorganisms (EMs) and Bioactivity-Driven Selection

**Figure 1:**
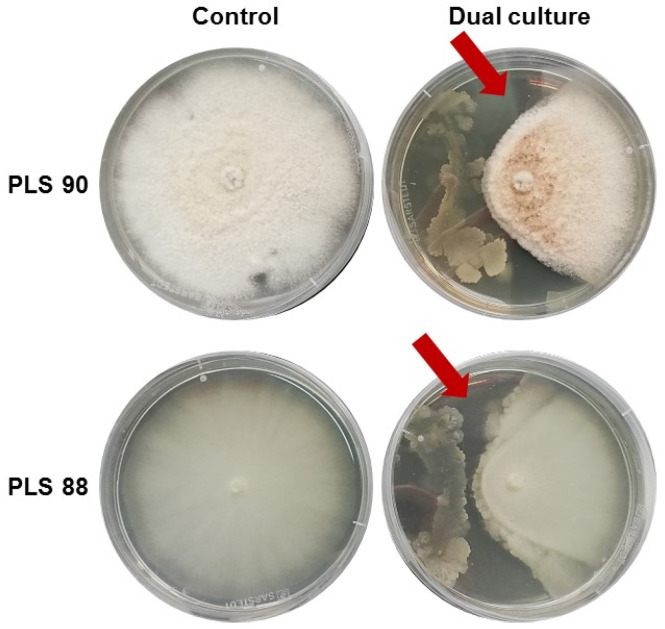
Confrontation bioassays for the bioactivity assessment of the olive tree bacterial isolate *Bacillus* sp. PTA13 against *Colletotrichum acutatum* species complex PLS 90 (wild type) and PLS 88 (resistant to PPPs) isolates. The formation of inhibition zone (red arrows) and the pattern of the fungal culture development were used as indicators of the bioactivity of the endophyte.

The applied protocol resulted in the isolation of 53 olive tree EMs, out of which 29 were bacteria and 24 fungi. The grouping of the latter was based on phenotypic characteristics, the growth rate of their cultures, and microscopic observations. The majority of EMs were isolated from roots (39), whereas a handful were isolated from leaves (5), fruits (7), and shoots (2). The selection of the microorganisms to be further investigated was based on their bioactivity against the target pathogenic fungus *C. acutatum* in confrontation bioassays, applying a bioactivity-driven approach (Figure 1 and Figure S1). Six bacteria that were isolated from roots exhibited strong antifungal activity, and among these isolates, the PTA13 exhibited the strongest inhibition, as indicated by the extended inhibition zone (Figure 1), and thus, it was selected to be further investigated in the present study.

### 2.2. The Bacillus sp. Is the Predominant Endophytic Microorganism (EM) of the Olive Tree Roots Studied

Applying PCR, the positions 27 and 534 of the bacterial 16S rRNA genes were amplified using universal primers. The reaction products were further subjected to electrophoresis (Figure S2). The appearance of bands in the samples of the EMs and the absence of corresponding bands in the negative control confirmed the successful amplification of the EM genomic DNA fragments. Additionally, the purification of the obtained DNA fragments was confirmed using a Nanodrop spectrophotometer ND-2000C (Thermo Scientific, Waltham, MA, U.S.A.). The fragments exhibited an A_260_/A_280_ ratio between 1.8 and 1.9, indicative of their quality, and were further diluted with nuclease-free water to a final concentration of 40 ng∙μL^−1^, which was used for sequencing. All six bacterial isolates that exhibited the highest bioactivity against *C. acutatum* (see Section 2.1) were identified as *Bacillus* sp. based on the results of sequencing (data not shown). Nonetheless, the *Bacillus* sp. PTA13 isolate, based on its superior bioactivity, was selected for further experimentation.

### 2.3. Isolation of the Bacillus sp. PTA13 Lipopeptides (LPs) and Deconvolution of Their Metabolite Composition Applying LC/ESI/MS/MS Analysis

Bacterial species that belong to the genus *Bacillus* are often characterized as microbial factories due to the vast number of structurally diverse metabolites that they produce; their variable bioactivities, including antimicrobial properties against phytopathogenic fungi; and their roles in their biology and survival [21,24,25]. Among the groups of secondary metabolites that *Bacillus* sp. produces is that of LPs [21,24,26]. Based on their unique properties and bioactivity [36], here, effort was made to isolate LP-enriched fractions from the *Bacillus* sp. PTA13 strain and to study their bioactivity against *C. acutatum*. The separation of the bioactive LP fraction from substances of the culture medium was based on previously described protocols applying acid precipitation of lipopeptides (LPs) [37,38]. Due to the relatively low yield of LPs (approximately 345 mg of precipitate was obtained from the acid precipitation of 1 L of culture medium), the above procedure was repeated several times in order to obtain a sufficient amount of the precipitate for further analysis and evaluation.

**Figure 2:**
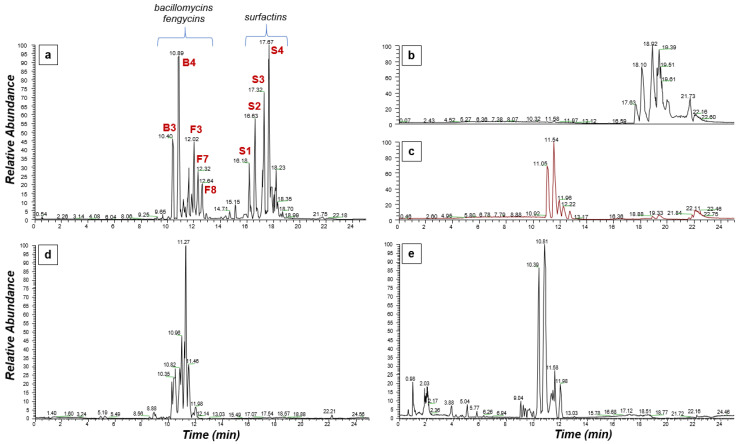
LC/ESI/MS total ion chromatograms (TICs) of *Bacillus* sp. PTA13 lipopeptides (LPs). TIC of (**a**) the total LP extract, (**b**) the surfactin-containing upper phase during liquid–liquid extraction, and (**c**) the corresponding lower phase that contains bacillomycins and fengycins, and the representative TIC of fractions derived from size-exclusion chromatography of the lower phase (see plot c) that contain (**d**) fengycins and (**e**) bacillomycins. Annotations of representative LPs are displayed (see Table 1).

The comprehensive deconvolution of the LP extract was based on the superior bioanalytical capacities of the Orbitrap analyzer that enabled the recording of metabolite features at under 2 ppm (Table 1). In the obtained total ion chromatogram (TIC) of the LP extract, two distinct groups of peaks are observed (Figure 2a), which correspond to the main families of LPs produced by *Bacillus* sp. PTA13. Specifically, the analysis of the acquired data revealed the presence of metabolites that belong to the three main categories of LPs, namely bacillomycins (subgroup of iturins), fengycins, and surfactins (Table 1). The first group of peaks (RT 10–13 min) contains metabolites of the bacillomycin and fengycin families, while the second (RT 16–19 min) contains metabolites of the surfactin group. Furthermore, low abundance metabolite features that possibly belong to various LPs were also recorded.

**Figure 3:**
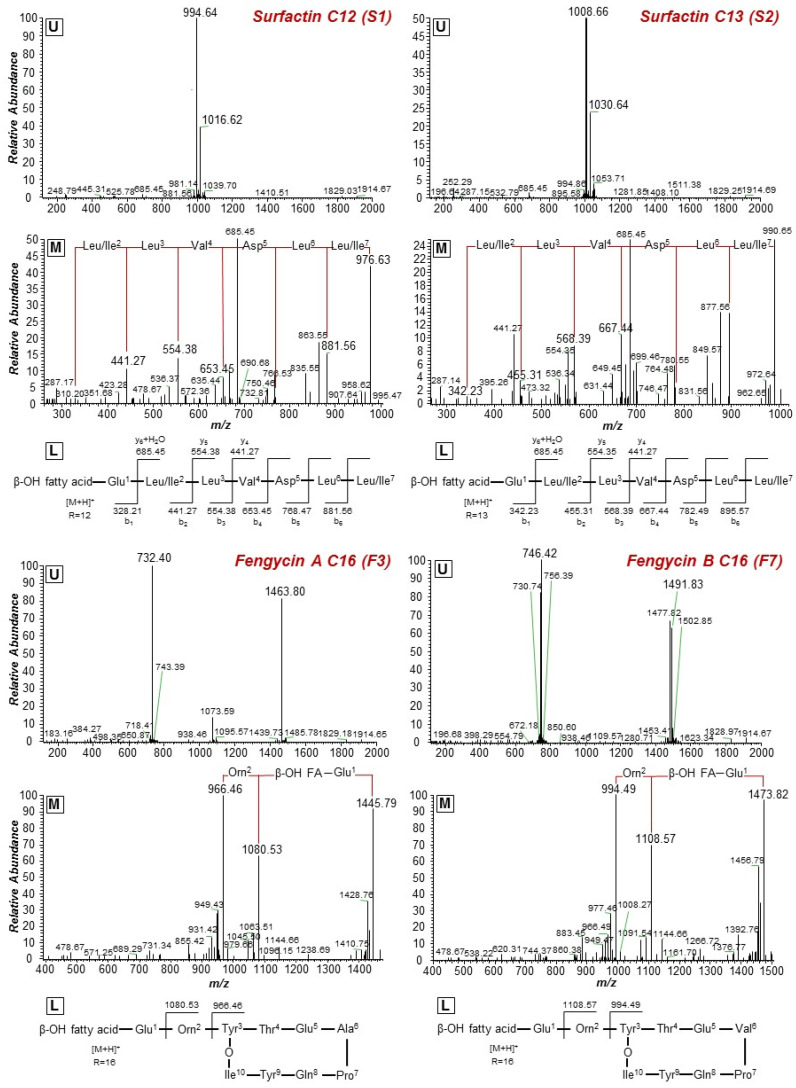
MS^1^ (**upper plots; U**) and MS^2^ (**middle plots; M**) of the annotated *Bacillus* sp. PTA13 lipopeptides (LP) surfactins C12 and C13, and fengycins A C16 and B C16. The fragmentation patterns were used for the identification of the LP (**lower plots; L**).

The major peaks of bacillomycins (four annotated LPs) were detected at RT 10.40, 10.89, 11.63, and 12.05 min, corresponding to [M+H]^+^ and [M+Na]^+^ adducts, while the major peaks of fengycins (eight annotated LPs, four fengycin A and four fengycin B) were detected at RT 11.20, 11.54, 11.54, 11.84, 12.02, 12.27, 12.32, and 12.64 min, corresponding to [M+H]^+^ and [M+H]^2+^ (double ionization charge) adducts. Finally, the major peaks for surfactins (four annotated LPs) were recorded at RT 16.18, 16.63, 17.32, and 17.67 min, corresponding to [M+H]^+^ and [M+Na]^+^ adducts (Table 1). For the tentative annotation of LPs in the analyzed fraction, a targeted approach was followed using MS/MS fragmentation patterns, an in-house built target library containing 76 LPs (Data Set S1), and information retrieved from the relevant literature. More specifically, the acquired fragmentation pattern of each substance allowed the deconvolution of the peptide backbone, with a clear fragmentation pattern for the amino acids (AAs) of the peptide moiety of surfactins (Figure 3 and File S1).

Based on such analysis, the 16 annotated LPs were categorized into four groups (Table 1), each one consisting of LPs with the same peptide backbone, differing in one methylene group (-CH_2_) in their fatty acid chain. Additionally, LPs of two different subgroups that both belong to fengycins (fengycins A and B) were annotated, differing in a single AA of their peptide ring. LPs of the fengycin A subgroup have the AA alanine (Ala) at position 6 of their peptide moiety, in contrast to those of the fengycin B subgroup, in which the AA valine (Val) is found at the corresponding position (Figure 3 and File S1).

### 2.4. Time-Course Study of the Bacillus sp. PTA13 Growth Rate and Its Lipopeptide-Producing Capacity

The obtained growth curve of the *Bacillus* sp. PTA13 (Figure S3a) is a typical sigmoid growth curve of bacterial cultures, which exhibits distinct phases. It was observed that under the experimental conditions set, the bacterial colony remains in the lag phase up to three hours following the media inoculation. Then, it proceeds to the exponential phase (up to nine hours post-inoculation) and, finally, to the stationary phase. The latter represents the phase during which the biosynthesis of metabolites increases. At this point, microorganisms uncouple the supply of energy and carbon from the purposes of their biomass production, diverting them to the biosynthesis of secondary metabolites [39].

Taking into account the data on the LP biosynthesis during the growth of *Bacillus* sp. PTA13 cultures, it seems that an early onset of the biosynthesis of surfactins is observed during the exponential growth phase (t = 6 h) (Figure S3b). The onset of the biosynthesis of the majority of bacillomycins and fengycins was recorded later at 9 h post-inoculation (Figure S3c–e). Another interesting finding regarding the biosynthesis of LPs is that the maximum production of surfactins is observed at 48 h, whereas that of bacillomycins and fengycins increases up to 72 h post-inoculation. Based on these data, acid precipitation was performed 48 h post-inoculation in order to obtain the LPs.

Based on the multiple roles of LPs in bacterial physiology and survival [24], the observed variation in the onset and course of production of the various groups of LPs could be plausibly indicative of their role and importance for *Bacillus* sp. PTA13. Thus, the early production of surfactins plausibly indicates their cornerstone role as quorum-sensing molecules [40] and their importance in the formation of biofilms and root colonization [41], attributes of vital importance for the survival and dispersal of the species. Similarly, bacillomycins can serve as signals by promoting the development of biofilm [42]. On the other hand, fengycins, in addition to their exceptional antimicrobial properties [43], seem also to inhibit the quorum sensing of bacteria [44], thus providing a substantial advantage during the antagonism of *Bacillus* sp. PTA13 with other species.

### 2.5. Isolation Protocols for the Separation of Bacillus sp. PTA13 Lipopeptide (LP) Groups When Applying Liquid–Liquid Extraction and Chromatography Techniques

The employment of methodologies with complementing capacities in the fractionation of complex extracts could facilitate not only the isolation and study of the major LP groups but also the detection of minor LPs, which are present at low abundances. In order to fractionate the initially obtained LP extract and separate the different LP groups, two isolation protocols were applied; the first aimed at the rapid separation of the three main LP groups, i.e., surfactins, bacillomycins, and fengycins, by using a combination of liquid–liquid extraction and size-exclusion chromatography, which exhibits improved efficacy in the separation of complex protein extracts [45]. The second was based on centrifugal partition chromatography (CPC) and aimed to improve fractionation of the LP extract. CPC exhibits improved capacity in the separation of complex mixtures and sample recovery [46] and facilitates the fractionation of a large number of LP mixtures and minor LPs.

#### 2.5.1. Selection of the Optimum Biphasic Solvent System

The selection of an optimum biphasic solvent system plays a cornerstone role in the successful separation of the components of an extract when applying liquid–liquid extraction as well as CPC analysis. Based on the results of the primary evaluation of the biphasic solvent systems being tested, systems 1–9 were not considered suitable to effectively fractionate the LP extract because of the low solubility of the extract (systems 1–4 and 7–9), the settling time (system 6), or the fact that the upper-to-lower phase ratio was not within the acceptable range (systems 2, 3, 5, and 9) (Table S1). On the contrary, systems 10–17 met the criteria and were further evaluated regarding the distribution of their components into the two phases by TLC (data not shown). Out of the eight systems being evaluated, systems 10 and 11 were disqualified due to the distribution of all the metabolites exclusively in the upper and lower phases, respectively. The remaining six systems (12–17) resulted in an improved chromatographic separation, and, thus, they were further analyzed by LC/ESI/MS/MS.

Based on the deconvolution of the obtained metabolite profiles (Figure S4), the solvent system 15 [n-Heptane: EtOAc: MeOH: H_2_O (2:3:2:3, *v*/*v*/*v*/*v*)] was selected as the optimum for the fractionation of the initially obtained LP extract. Using this system, the separation between surfactins, which are distributed in the upper phase, from bacillomycins and fengycins, which are distributed mainly in the lower phase, can be achieved robustly in a one-step process, which represents a great advantage. On the other hand, the biphasic system 13 [n-Heptane: EtOAc: MeOH: H_2_O (1:3:1:3, *v*/*v*/*v*/*v*)] presented a satisfactory distribution of the individual LP and, thus, was chosen for the CPC analysis of the LP extract.

#### 2.5.2. Rapid Separation of the Three Major Lipopeptide (LP) Groups (Surfactins, Bacillomycins, Fengycins) Using Liquid-Liquid Extraction and Size Exclusion Chromatography

Liquid–liquid extraction was employed for the initial separation of the major LP groups, such as surfactins, bacillomycins, and fengycins. It was observed that surfactins were recovered from the upper phase of the biphasic solvent system 15 [n-Heptane: EtOAc: MeOH: H_2_O (2:3:2:3, *v*/*v*/*v*/*v*)] (Figure 2b), whereas bacillomycins and fengycins were recovered from the lower (Figure 2c). Applying such a protocol to 200 mg of the LP extract, surfactins were efficiently isolated with a yield of 90.6 mg, while 92.9 mg of bacillomycins and fengycins was recovered from the lower phase.

The lower phase containing bacillomycins and fengycins was further subjected to size-exclusion chromatography for their further separation, which was based on the difference between their molecular weights. In total, 60 fractions of 2 mL each were obtained. The chromatographic separation was assessed applying TLC, which confirmed the anticipated separation between the fengycin and bacillomycin groups (Figure S5). More specifically, fengycins, which have a higher molecular weight, were firstly eluted from the Sephadex column, with the highest abundance recorded in the fractions 19–31. Bacillomycins were eluted next, detected in the fractions 37–60. Such separation can be clearly observed following the spraying of the TLC plate with ninhydrin solution, since both LP classes contain primary amino groups in their peptide backbone, which react with ninhydrin to form purple-colored products (Figure S5). The LC/ESI/MS analysis of the fractions that were obtained by the size-exclusion chromatography confirmed the successful separation between the two classes of LPs. Representative chromatograms of the fengycin and bacillomycin fractions are displayed in Figure 2d,e, respectively. Based on the results of the LC/ESI/MS analysis, an appropriate combination of the fractions of the size-exclusion chromatography was performed resulting in a total of five combined fractions, with fraction 3 containing mainly fengycins and fraction 5 containing mainly bacillomycins.

Additionally, further fractionation of the lower phase of the liquid–liquid extraction revealed the presence of metabolites that are present at low abundances in the total LP extract and that were not identified during the initial analysis. More specifically, two additional bacillomycins were identified, which consisted of the same peptide ring as the abovementioned (Section 2.3), while differing in the length of their fatty chain, having one (fatty acid chain with 13 C) and two (fatty acid chain with 12 C) less methylene groups, respectively, than the first identified bacillomycin. In fraction 37, which had the highest content of these two bacillomycins compared to the rest, their major peaks were detected at RT 9.23 and 9.69 min, which correspond to [M+H]^+^ and [M+Na]^+^ adducts (Table 1).

#### 2.5.3. Application of Centrifugal Partition Chromatography (CPC) Resulted in the Detection and Separation of Low and High Abundance Lipopeptides (LPs)

The capacity of CPC to separate the different groups of the LPs was assessed using the biphasic system n-Heptane: EtOAc: MeOH: H_2_O (1:3:1:3, *v*/*v*/*v*/*v*). Initially, 500 mL of the mobile phase was used during the elution step, and 50 fractions were collected. Then, using 250 mL of the stationary phase during the extrusion of the column-retained content, 25 additional fractions were collected. The whole process lasted 95 min, and 75 fractions of 10 mL were obtained in total. The LP content of the obtained fractions was initially qualitatively assessed by performing TLC as described below. Based on the obtained chromatograms, the fractions exhibiting a similar chemical composition were combined and evaporated to dryness. The composition of the combined fractions was monitored by applying TLC, and the comprehensive chemical analysis was performed using LC/ESI/MS as described in Section 3.9. To the best of our knowledge, there are no previous reports on the separation of *Bacillus* LP employing CPC analysis.

In the TLC chromatograms of the combined fractions (data not shown), the fractionation achieved by the CPC analysis was further confirmed. The 14 obtained fractions differ in their chemical composition, with the initial ones exhibiting, as expected, high content of the most polar LPs of the total extract, bacillomycins and fengycins, while the following ones have a high concentration of surfactins. More specifically, it seems that bacillomycins are more abundant in the first CPC fractions (combined fractions 1 and 1a), fengycins are mainly present in the combined fractions 2–7, and the surfactins are mainly present in the fractions 10–13.

**Figure 4:**
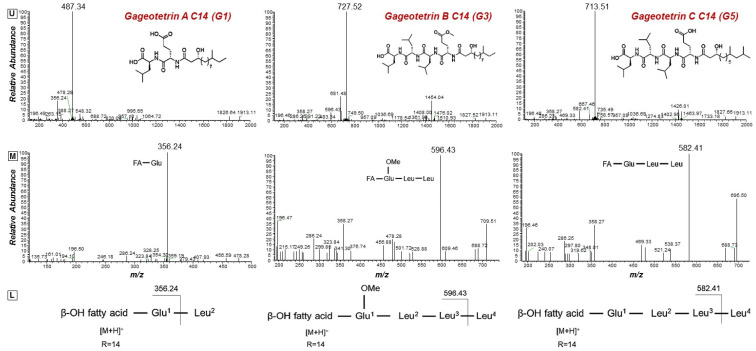
MS^1^ (**upper plots; U**) and MS^2^ (**middle plots; M**) of the *Bacillus* sp. PTA13 LP Gageotetrins A–C. The fragmentation patterns were used for the annotation of the LP (**lower plots; L**).

The results of the LC/ESI/MS analysis of the combined CPC fractions revealed a gradual elution of the LP groups of the total extract (Figure S6). The CPC analysis was not efficient in separating the various LP groups; nonetheless, fractions of variable composition were obtained. These fractions were further assessed regarding their antifungal activity. Such bioassays could additionally provide insights into the contribution of the individual LP groups to the toxicity of the total fraction. In addition, fractions enriched in low abundance metabolites, which were not detected and identified performing LC/ESI/MS analysis of the total LP extract, were obtained. Specifically, the fractions 10 and 11 contained high concentrations of two low abundance groups of LP, namely gageotetrins and bacilotetrins, which include small LPs and whose peptide backbone consists of two to four AAs. The gageotetrin family consists of linear LP exhibiting broad-spectrum antimicrobial properties [47], with gageotetrin A being the smallest LP group that occurs in nature, since they are composed of only two AAs and an aliphatic chain (Figure 4). The other two groups of gageotetrins (B and C) are structurally similar, with the only difference being the presence of a methoxy group (-OCH_3_) attached to the glutamic acid of gageotetrin B (Figure 4). On the contrary, bacilotetrins are cyclic molecules with four AAs in their peptide moiety and exhibit antistaphylococcal activity [48].

In fraction 11, which exhibited the highest concentration of the aforementioned LP, gageotetrins were detected at RT 13.95 and 14.49 (gageotetrin A), 16.14 (gageotetrin B), and 14.69 and 15.61 (gageotetrin C) min, which correspond to the [M+H]^+^ adduct. In addition, bacilotetrins were detected at RT 17.01 (bacilotetrin A) and 17.43 (bacilotetrin B) min, the [M+H]^+^ adduct (Table 1). The identification of the above LP was performed by HRMS/MS analysis (Figure 4).

### 2.6. The LP Extract of Bacillus sp. PTA13 and Its Fractions Are Highly Toxic to Both the Susceptible and Resistant to Fungicides Isolates of the Colletotrichum Acutatum Species Complex

**Figure 5:**
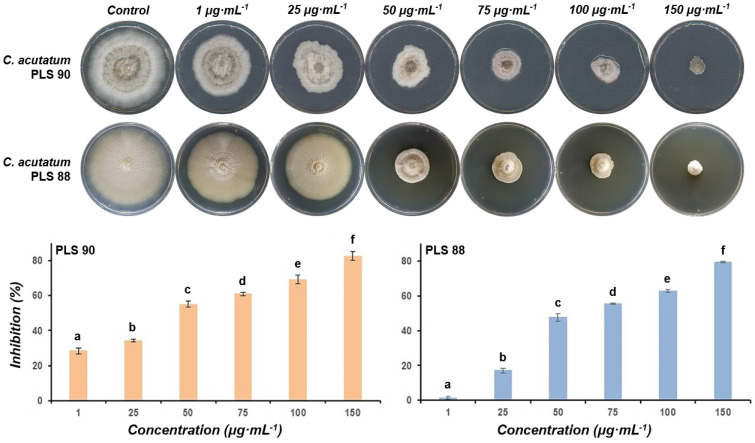
Effect of the total lipopeptide extract of *Bacillus* sp. PTA13 on the radial growth of cultures of the olive tree pathogenic *Colletotrichum acutatum* species complex PLS 90 (wild type) and PLS 88 (resistant to PPPs) isolates. Images of inhibition and observations were taken 17 days post-treatment with N = 3 biological replications. The letters (a–f) above the columns indicate statistical differences between the treatments for each isolate, performing the Tukey HSD test (*p* > 95%).

**Figure 6:**
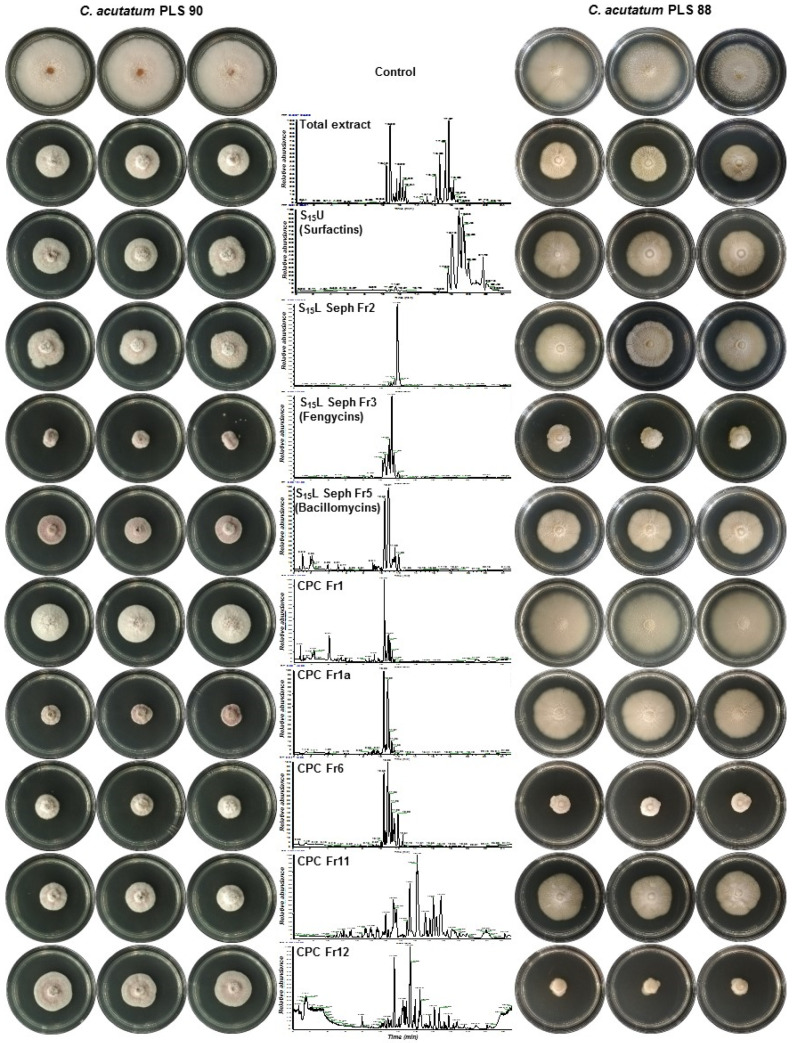
Effect of various fractions of the *Bacillus* sp. PTA13 total lipopeptide extract on the radial growth of the *Colletotrichum acutatum* species complex isolates PLS 90 (wild type) and PLS 88 (resistant to PPPs). The fractions were applied at concentrations equal to the EC_50_ of the total LP extract for PLS 90 (27 μg mL^−1^) and PLS 88 (63 μg mL^−1^). Observations were taken 10 days post-treatment, and three biological replications were performed per treatment (CPC; Centrifugal Partition Chromatography, L; Lower Phase, S; Biphasic Solvent System, Seph; Sephadex, U; Upper Phase).

**Figure 7:**
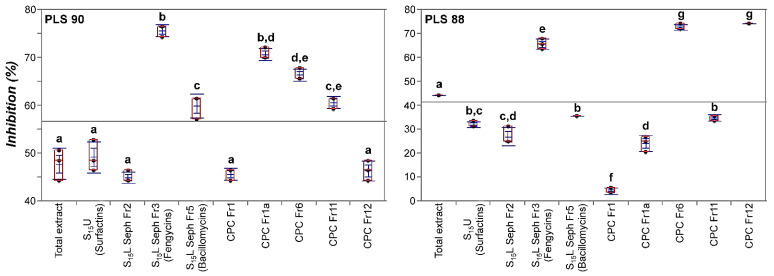
Effect of various fractions of the *Bacillus* sp. PTA13 lipopeptide extract on the radial growth of the *Colletotrichum acutatum* species complex isolates PLS 90 (wild type) and PLS 88 (resistant to PPPs) displayed using box plots. The fractions were applied at concentrations equal to the EC_50_ of the total LP extract for PLS 90 (27 μg mL^−1^) and PLS 88 (63 μg mL^−1^). Three replications were performed per treatment and the different letters above boxes designate statically significant differences performing the Tukey HSD test (*p* > 95%) (CPC; Centrifugal Partition Chromatography, L; Lower Phase, S; Biphasic Solvent System, Seph; Sephadex, U; Upper Phase).

The issue of the development of pest and pathogen resistance to PPPs is among the major challenges that the agrochemical sector is currently facing [14,49]. Within this context, the exploitation of new sources of bioactivity for applications in plant protection seems to be an alternative with high potential to combat the issue. Here, the LP extract of *Bacillus* sp. PTA13 and its fractions containing mainly LPs of the surfactin, bacillomycin, and fengycin groups exhibited a variable toxicity to the two *C. acutatum* species complex isolates being tested. The LP extract proved to be toxic to both *C. acutatum* isolates, and, interestingly, the resistant-to-fungicides isolate PLS 88 was less susceptible (median effective concentration, EC_50_ = 63 μg mL^−1^) than the PLS 90 (EC_50_ = 27 μg mL^−1^) (Figure 5). Based on the obtained evidence and the complexity of the extract’s composition, such selectivity cannot be explained. The main families of LPs present in the extract (e.g., surfactins, bacillomycins, and fengycins) could either interfere in the plant–pathogen interactions or be directly toxic to microorganisms mainly by acting on their membranes via a pore-forming mechanism [27,50]. Based on the abovementioned, it is plausible to suggest that differences in the structure/composition between the membranes of the PLS 88 and PLS 90 isolates could be responsible for the observed phenotypes. Such results are in complete alignment with previous reports on the antimicrobial activity of *Bacillus* metabolites [51], confirming the bioactivity of the total LP extract of *Bacillus* sp. PTA13 against the olive tree phytopathogen, including the isolate with proven resistance to commercial fungicides applied in olive tree plant protection [34]. The surfactin fraction exhibited toxicity to the PLS 90 equal to that of the total LP extract, while for the PLS 88, a reduced toxicity was recorded (Figure 6 and Figure 7). The fraction is a mixture of surfactins, which are molecules with well-established bioactivity, including, among others, antifungal, antiviral, and insecticidal activities [52]. The antimicrobial properties of surfactins have been mainly attributed to membrane permeabilization [51,52]. The abovementioned confirm the bioactivity of the *Bacillus* sp. PTA13-produced surfactins against the phytopathogen being tested; however, for the resistant strain, it seems that they exhibit a reduced toxicity, which is lower than that of fengycins and bacillomycins. The fengycin-enriched fractions proved to be the most bioactive against the phytopathogens, exhibiting inhibition of more than 60% compared to the untreated (Figure 6 and Figure 7). Fengycins are a group of LPs to which the bioactivity of *Bacillus* sp. against phytopathogenic fungi is largely attributed [23], a report that is in agreement with our findings. Although there is no solid evidence on their MoA, at low concentrations, it seems that they cause the formation of single-ion channels, whereas high concentrations lead to their solubilization [23,53,54,55]. Based on the obtained results, it seems that the two fengycin-enriched fractions are almost equally toxic to both isolates being tested, which represents an advantage from a plant protection perspective. Bacillomycins exhibited a moderate toxicity to both isolates, with PLS 90 being more sensitive than PLS 88 (Figure 6 and Figure 7). This fraction was obtained by size-exclusion chromatography and contains various bacillomycins, whose antimicrobial activity has been previously documented [56], confirming our observations.

To our surprise, the only fraction that showed selective toxicity to the resistant PLS 88 isolate was the fraction 12 of CPC (Figure 6 and Figure 7). This is a very interesting finding, since the primary aim of plant protection strategies is to combat resistant plant pathogen populations. The fact that this fraction is composed of minor linear and cyclic LPs (e.g., gageotetrins and bacilotetrins) further supports the notion of our approach to mine in depth the total LP extract toward the discovery of such bioactivity. These compounds are known for their improved antimicrobial activity [47,48,57], which is in agreement with our findings. Gageotetrins are toxic to phytopathogens such as *Phytophthora capsici*, which causes the disease late blight, but on the other hand, they are not toxic to human cancer cell lines. [47]. Furthermore, their inhibitory effect on hyphal growth and conidial germination has recently been reported for *Magnaporthe oryzae*, which causes the disease wheat blast [57]. It is plausible that both the peptide chain and the fatty acid of gageotetrins largely determine their bioactivity. The family of bacilotetrins contains LPs with antimicrobial properties, which, similarly to gageotetrins, exhibit no cytotoxicity to human cancer cell lines. Taking the above results into consideration, the current study demonstrates the potential of the EMs as a significant source of bioactive compounds exhibiting toxicity to the devastating olive tree phytopathogen *C. acutatum*, including strains resistant to commercially important PPPs.

## 3. Materials and Methods

### 3.1. Plant Material and Sample Collection

Olive tree tissues (roots, shoots, fruits, and leaves) of the variety “Koroneiki”, one of the most commercially important oil-producing Greek cultivars, were sampled from olive orchards of the Lakonia region (prefecture of Peloponnese) in southern Greece (Figure S7). Samples were collected from healthy olive trees of orchards, to which no prior treatment with conventional PPPs and/or biological agents had been performed. These orchards are also located far from cultivated areas, to which such products are regularly applied. Specific attention was given to collect samples from non-infected plant tissues, including aboveground and underground tissues, such as branches, leaves, fruits, and parts of the root or root hairs. The samples were collected in plastic bags, stored at 4 °C, and further processed within 24 h.

### 3.2. Isolation and Cultivation of Olive Tree Endophytic Microorganisms (EMs)

The collected samples of olive tree roots and shoots were initially cut into small pieces (approximately 5 cm in length), whereas the leaves and fruits were processed without prior sectioning. The samples were surface sterilized to avoid possible isolation of epiphytic microorganisms, according to previously described protocols [17,58,59]. Briefly, the process was performed by stepwise immersion of the tissues in 100% (*v*/*v*) ethanol (1 min), 5% (*v*/*v*) sodium hypochlorite (4 min), and 100% (*v*/*v*) ethanol (30 s) and, finally, rinsing them four times with sterile deionized water, for 1 min each time. Imprints of the sterilized tissues were then taken by placing them for a few seconds onto the surface of Potato Dextrose Agar (PDA, Becton, Dickinson and Company, Sparks, MD, USA) in 9-cm-diameter Petri plates to ensure the efficacy of the applied sterilization protocol. Finally, the sterilized tissues were cut into small pieces, placed on PDA, and incubated at 22 °C, in the dark. Observations were made regularly, and upon the development of microorganisms (fungi and/or bacteria), a small portion of the colonies was transferred to new PDA plates to obtain pure cultures, which were incubated as described above. Pure EM cultures were obtained by four rounds of re-isolation by peaking single colonies via streaking in the case of bacteria or by transferring hyphae from the edges of the growing fungal colonies. All handling was performed aseptically in a horizontal laminar flow hood.

For the regular maintenance of the obtained EMs, they were next cultivated in Petri plates (9 cm diameter) containing PDA and incubated in the dark at 22 °C. Sub-culturing was performed in 15-day intervals. Additionally, bacterial cultures were grown in lysogeny broth (LB) medium under continuous agitation (120 rpm) in an orbital incubator at 28 °C in the dark in order to evaluate their growth rate and also to scale up the production of LPs. For the long-term maintenance of the EMs, 5-mm-diameter mycelial plugs or bacterial suspensions from freshly prepared cultures were transferred to 1.5 mL Eppendorf tubes containing glycerol:phosphate-buffered saline (PBS) (1:1, *v*/*v*) and stored at −80 °C.

### 3.3. Assessment of the Bioactivity of the Olive Tree Endophytes and That of Lipopeptide (LP) Extract/Fractions to Colletotrichum Acutatum Species Complex Isolates

The bioactivity of the obtained EM isolates against *C. acutatum* was assessed by performing the plate confrontation assay [60]. As the target species, the *C. acutatum* strains PLS 88 and PLS 90 were used, the former exhibiting resistance to fungicides and the latter being sensitive [34]. Because of the slow growth rate of *C. acutatum*, the pathogen was initially inoculated by transferring a 5-mm-diameter mycelial plug from the edges of a 14-day old culture to the center of PDA plates. Three days post-inoculation, the EMs were inoculated symmetrically on both sides of the *C. acutatum*-growing colony. The fungal endophytes were inoculated by using mycelial plugs, whereas bacterial endophytes were inoculated by using inoculum (3 μL) from cultures growing in LB medium. The bacterial cultures grew for 8 h (exponential phase), and inocula were taken when their OD_600_ was 0.6. Observations of the dual cultures were made regularly until the formation of inhibition zones, which was used as the indication of bioactivity, or until the cultures of the confronting microorganisms overlapped.

For the assessment of the LP extract’s bioactivity against both isolates of the phytopathogen, starter cultures were prepared in PDA and incubated for 14 days, as described above. Initially, for the estimation of the EC_50_ of the total LP extract, PDA plates (9 cm diameter) were amended with a range of concentrations of the LP extract (1, 25, 50, 75, 100, and 150 μg mL^−1^) and were inoculated by transferring 5 mm mycelial plugs to their center from the edges of the starter cultures. The assessment of the toxicity of the LP extract was based on its effect on the radial growth of fungal cultures, as measured by the software ImageJ [61]. Observations were made at 24 h intervals, and statistical analyses were performed using the statistical software JMP v.15 (SAS Institute Inc., Cary, NC, USA) (Tukey HSD test, *α* = 0.05). Three biological replications were performed per treatment, and the final observation was made seventeen days post-treatment. In a second step, following a similar protocol, the obtained LP fractions from the performed extraction and chromatography techniques were applied to both *C. acutatum* isolates at concentrations equal to the corresponding EC_50_ values. Bioassays were performed in PDA in 5 cm diameter Petri plates, and the final observation was made ten days post-treatment.

### 3.4. Molecular Identification and Characterization of Endophytic Bacteria

Based on the results of the confrontation bioassays (Section 2.1), the most bioactive endophytic bacterial isolates were chosen to be further identified and studied. The bacteria were cultivated in LB medium as described above (Section 3.2) for 24 h, when the culture reached a density of approximately 10^8^–10^9^ colony-forming units (CFU)∙mL^−1^. For the extraction of bacterial DNA, a previously described protocol was followed with minor modifications [17]. Briefly, the bacterial cultures were collected and centrifuged (10 min, 5000 rpm, 3 °C), and the supernatant was discarded.

For the DNA extraction of the resulting cell pellet, the PureLink Microbiome DNA Purification Kit (Thermo Scientific, San Jose, CA, USA) was used according to the manufacturer’s instructions. The concentration and purity of the extracted DNA were assessed using a Nanodrop spectrophotometer ND-2000C (Thermo Scientific), and the concentration of the extract was finally adjusted to 40 ng∙mL^−1^ using double-distilled sterilized water. The universal primers 27F and 534R, which amplify the positions 27 and 534 of the bacterial 16S rRNA genes, respectively, were used [17]. The targeted genes were amplified (polymerase chain reaction-PCR, Applied Biosystems Veriti^TM^ 96-Well Thermal Cycler, Thermo Scientific) using the selected primers. The reactions were carried out using the DreamTaq Green PCR Master Mix (2×) (K1081, Thermo Scientific) in a final volume of 50 μL. For quality control purposes, a negative control without DNA ran concurrently with the reaction. The thermal cycles were as follows: denaturation at 95 °C for 4 min, 35 cycles of 95 °C for 30 s, 56 °C for 30 s, and 72 °C for 1 min, ending at 8 °C. The amplicon was visualized by electrophoresis (1% *w*/*v* agarose gel) using LumiBIS 1.4 (DNR Bio-Imaging Systems Ltd., Neve Yamin, Israel) and the molecular marker GeneRuler 100bp DNA ladder (Thermo Scientific). The amplified PCR products were purified using NucleoSpin^®^ Gel and a PCR Clean-up Kit (Macherey-Nagel, Duren, Germany) and were sequenced (Cellular and molecular immunological applications, CeMIA SA, Larissa, Greece). The results were blasted using the NCBI’s BLASTn software, and the top hits were used to identify the bacterial isolates at the genus level, where applicable.

### 3.5. Isolation of Lipopeptides (LPs) from Liquid Cultures of the Endophytic Bacillus Strain PTA13

In order to isolate the LPs produced by the endophytic *Bacillus* strain PTA13, 20 mL of liquid LB medium was initially inoculated with bacterial suspension from a glycerol stock culture stored at −80 °C and incubated as mentioned above (Section 3.2). Two days post-inoculation, 3 mL of the culture was transferred to 500 mL Erlenmeyer flasks containing 300 mL of LB. The new cultures were incubated for 48 h under the aforementioned conditions. The bacterial cells were then removed by centrifugation (8500 rpm, 20 min, 4 °C), and the supernatant was collected. The separation of the LPs contained in the supernatant was performed by acid precipitation following previously described protocols with minor modifications [37,38]. Briefly, a hydrochloric acid solution (HCl 6M) was added to the cell-free supernatant to pH = 2, followed by overnight storage at 4 °C. The resulting precipitate was collected by centrifugation (8500 rpm, 20 min, 4 °C), and the obtained LPs were further purified by ultrasound-assisted extraction using 100 mL of a CHCl_3_:MeOH (2:1, *v*/*v*) mixture [62,63]. The extraction was repeated 3 times for 30 min each, and the collected extract was filtered and evaporated to dryness using a rotary evaporator (Buchi Rotovapor R-210; Buchi, Inc., Flawil, Switzerland). Due to the low yield of the bacterial culture in LP, the overall procedure was repeated several times to obtain a sufficient amount of the LP extract to be used in the bioassays. Indicatively, 1 L of bacterial culture yielded approximately 345 mg of LP extract.

### 3.6. Time-Course Study of the Bacillus sp. PTA13 Growth Rate and Its Lipopeptide-Producing Capacity

The aim of the experimentation was the discovery of the optimal time of the production of LPs in order to maximize the yield, as well as the investigation of the biosynthesis of the various LPs by the bacterium in the time-course study, under the conditions set. The culture of the bacterium was performed as described in Section 3.2. In total, three 500 mL conical flasks containing 300 mL of LB medium were inoculated (t = 0). The cultures were incubated for 72 h, and the monitoring of LP biosynthesis was performed by sampling 1 mL from each conical flask at the following time points: t = 0, 3, 6, 9, 12, 24, 30, 36, 48, 54, and 72 h. For each sample, the OD_600_ was recorded using a Uvikon 922 (Kontron Instruments, Ismaning, Germany) spectrophotometer in order to monitor the growth rate of the culture and estimate the corresponding growth curve.

For the analysis of LP production, the samples were centrifuged, and the supernatants were transferred to Eppendorf tubes. Then, the samples were concentrated to dryness and redissolved in 1 mL H_2_O (MS grade). Finally, dilution (1/10) was performed by adding 900 μL MeOH:H_2_O (50:50, *v*/*v*) to 100 μL of the samples. An LC-Hybrid LTQ-Orbitrap Discovery platform (Thermo Scientific) platform was employed in the analyses (see analytical conditions in Section 3.9). The recorded TICs (11 time points × 3 replications = 33 chromatograms) were analyzed using the software Xcalibur (Thermo Scientific).

### 3.7. Fractionation of the Bacillus sp. PTA13 Lipopeptide (LP) Extract by Applying Liquid–Liquid Extraction and Chromatography Techniques

#### 3.7.1. Assessment of Biphasic Solvent Systems

The fractionation of the LP extract carried out by applying liquid–liquid extraction and CPC required the prior selection of the appropriate biphasic solvent system. Therefore, seventeen biphasic solvent systems (Table S1) were prepared and assessed regarding their settling time and phase ratios, as well as the solubility of the extract following a previously described protocol with minor modifications [64]. Briefly, a portion of the LP extract (10 mg) was dissolved in 5 mL of each system in a 10 mL glass tube, followed by vortexing and, finally, equilibration of the biphasic system. The solvent systems that exhibited a settling time of less than 1 min, an upper-to-lower phase ratio of approximately 1, and a high extract solubility were further evaluated by performing TLC and LC/MS analyses in order to determine the distribution of the components of the extract to the two phases. Following the equilibration of the optimum biphasic systems, aliquots (1 mL) of each phase were evaporated to dryness, redissolved in 1 mL MeOH, and subjected to TLC and LC/MS analyses.

#### 3.7.2. Liquid–Liquid Extraction

The liquid–liquid extraction of the LP extract was performed in a 250 mL separatory funnel using the biphasic solvent system n-Heptane/EtOAc/MeOH/H_2_O (2:3:2:3, *v*/*v*/*v*/*v*). Initially, 200 mg of the extract was dissolved in 10 mL of the solvent system and was added to the funnel to a final volume of 200 mL. The resulting solution was shaken vigorously and then allowed to equilibrate for the separation between the two phases. The upper and lower phases were collected separately in pre-weighed spherical flasks and were evaporated to dryness.

#### 3.7.3. Size-Exclusion Chromatography

The lower phase that was obtained by performing liquid–liquid extraction of the LP extract (Section 3.7.2) was further subjected to size-exclusion chromatography, by which the components of an extract are separated based on their molecular weight and adsorption capacity. A portion (50 mg) was dissolved in 1.5 mL of MeOH, and the resulting solution was loaded and pumped through a Sephadex LH-20 column (25–100 μm, Sigma-Aldrich Ltd., Steinheim, Germany) using MeOH as the eluent. The obtained 2 mL fractions were analyzed using TLC and LC/ESI/MS in order to assess their complexity and annotate their components. Based on the results of such analyses, the fractions were appropriately combined (e.g., fractions containing the same LP groups were combined).

#### 3.7.4. Semi-Preparative Centrifugal Partition Chromatography (CPC) Analysis

The CPC analysis of the LP extract was performed by employing an FCPC apparatus (Kromaton, Anonay, France) equipped with a 200 mL semi-preparative chromatographic column. The analysis was carried out in elution extrusion mode, using the biphasic solvent system n-Heptane/EtOAc/MeOH/H_2_O (1:3:1:3, *v*/*v*/*v*/*v*), the upper phase of which served as the stationary phase, while its lower phase served as the mobile one. In the first step, the stationary phase was added in the column in ascending mode (flow rate 10 mL∙min^−1^ and rotation speed 250 rpm) using a preparative Ecom ECP2000 pump (Prague, Czech Republic). Then, the mobile phase was pumped into the column in descending mode, applying the same flow rate and increasing the rotation speed to 600 rpm, in order for the biphasic system to equilibrate inside the column. Following the equilibration of the solvent system, the retention volume of the stationary phase was estimated. The latter was 100 mL and the corresponding Sf value 50%. A portion of the dry LP extract (300 mg) was dissolved in 8 mL of the biphasic system (upper: lower phase, 1:1, *v*/*v*), and it was injected on the column. During the elution step, 400 mL of the mobile phase was used at a flow rate of 10 mL∙min^−1^ and a rotation speed of 600 rpm (descending mode). Extrusion of the content of the column was performed using 200 mL of the stationary phase (descending mode). The collection of the fractions (10 mL) was carried out using a C6-60 Buchi collector (Flawil, Switzerland) at a rate of 1 fraction per min.

### 3.8. Thin-Layer Chromatography (TLC) Analysis of the Total Lipopeptide (LP) Extract and the Obtained Lipopeptide Fractions

TLC of the LP extract and its fractions was performed on aluminum TLC plates (TLC silica gel 60 F_254_ 20 × 20 cm, Merck, Darmstadt, Germany) using the solvent system CHCl_3_/MeOH/H_2_O (70:26:4, *v*/*v*/*v*) as the eluent. The TLC chromatograms were initially observed with UV light (254 and 366 nm) using a CAMAG TLC Visualizer and processed with the software visionCATS and winCATS. Additionally, the plates were treated with the chemical reagent ninhydrin, which is widely used for the detection of amino groups in analyzed samples. Ninhydrin reacts with primary amino groups to form purple-colored products (Ruhemann’s purple) [65]. The preparation of the reagent was performed by adding 0.2 g of ninhydrin to 100 mL of EtOH (0.2% *w*/*v*). The solution was sprayed onto the plates, followed by heating at 100 °C until the appearance of spots.

### 3.9. Ultra-High-Performance Liquid Chromatography–High-Resolution MS/MS (LC-HRMS/MS) Analysis of the Total Lipopeptide (LP) Extract and the Obtained Lipopeptide Fractions

For the deconvolution of the metabolite composition of the total LP extract and that of the obtained LP fractions, an H-Class Acquity LC system (Waters, Milford, CT, USA, San Jose, CA, USA) coupled to an LTQ-Orbitrap XL hybrid mass spectrometer (Thermo Scientific) was used. The platform was equipped with a quadrupole linear ion trap, an Orbitrap electrostatic Fourier transform mass spectrometer (FTMS) with an electrospray ionization (ESI) probe. Solutions of the dry extracts (200 μg mL^−1^) were prepared by dissolving them in 1 mL of MeOH:H_2_O (1:1, *v*/*v*) (MS grade). A Fortis C18 column (2.1 m × 100 µm I.D, 1.7 µm film thickness) was used for the chromatographic separation. The column temperature was kept at 40 °C, and the pressure ranged from 3500 to 4000 psi. The elution system consisted of water acidified with 0.1% formic acid (A) and acetonitrile (B) in the following gradient mode: 0–2 min 2% B, 2 to 18 min from 2% to 100% B, 18 to 20 min 100% B, 20–21 min from 100% to 2% B, and 21 to 25 min 2% B. The flow rate was set at 400 μL∙min^−1^ and the injection volume at 10 μL. Ionization was carried out in positive ion mode (ESI+). The mass spectrometric parameters were as follows: capillary temperature 350 °C; sheath gas 40 units; aux gas 10 units; capillary voltage 30 V; and tube lens 100 V. Data were recorded in full scan mode from 115 to 2000 *m/z*, and HRMS/MS experiments were carried out with a data-dependent method with collision energy 35.0% (q = 0.25). All experimental events were controlled using the software Xcalibur v.2.2.

## 4. Conclusions

The discovery of novel, alternative sources of bioactivity is necessary in order to address the challenges that the agrochemical sector is facing. Here, applying a bottom-up approach, the olive tree endophytic *Bacillus* sp. PTA13 was discovered as a potential customized plant protection agent of olive tree with bioactivity against the pathogenic fungus *C. acutatum*, which causes the devastating disease olive anthracnose. In addition to the potential use of the bacterium per se in plant protection, the isolated LP fractions (surfactins, bacillomycins, and fengycins) exhibited antifungal activity. Furthermore, there is increasing evidence indicating the ability of such LPs to trigger plants’ defense mechanisms. The results highlight the potential of olive tree EMs as alternative plant protection agents to the currently applied conventional and biological control agents, and their utilization as a rich source of bioactive compounds for further implementation in plant protection. The proposed pipeline is highly likely to become the primary plant protection strategy in the near future for customized plant protection and a major research priority for the agrochemical industry, integrating well with the concept of sustainability and the “farm to fork” approach.

## Figures and Tables

**Table 1:** Annotated lipopeptides (LPs) of the olive tree endophytic isolate *Bacillus* sp. PTA13. LPs were recorded at an average *Δppm* < 2 employing an LC-Hybrid LTQ-Orbitrap Discovery platform.

Peak	Mass (Da)	Rt (min)	Assignment	Molecular Formula	Sequence
Surfactins					
S1	994.6423	16.18	C12[M+H]^+^	C_50_H_87_N_7_O_13_	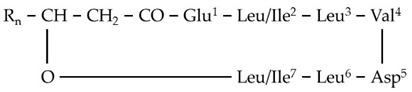
	1016.6233		C12[M+Na]^+^		
S2	1008.6575	16.63	C13[M+H]^+^	C_51_H_89_N_7_O_13_	
	1030.6389		C13[M+Na]^+^		
S3	1022.6728	17.32	C14[M+H]^+^	C_52_H_91_N_7_O_13_	
	1044.6539		C14[M+Na]^+^		
S4	1036.6890	17.67	C15[M+H]^+^	C_53_H_93_N_7_O_13_	
	1058.6700		C15[M+Na]^+^		
Bacillomycins D					
B1	1003.5117	9.23	C12[M+H]^+^	C_46_H_70_N_10_O_15_	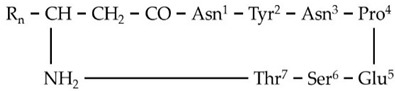
	1025.4904		C12[M+Na]^+^		
B2	1017.5267	9.69	C13[M+H]^+^	C_47_H_72_N_10_O_15_	
	1039.5086		C13[M+Na]^+^		
B3	1031.5400	10.40	C14[M+H]^+^	C_48_H_74_N_10_O_15_	
	1053.5211		C14[M+Na]^+^		
B4	1045.5540	10.89	C15[M+H]^+^	C_49_H_76_N_10_O_15_	
	1067.5359		C15[M+Na]^+^		
B5	1059.5710	11.63	C16[M+H]^+^	C_50_H_78_N_10_O_15_	
	1081.5527		C16[M+Na]^+^		
B6	1073.5866	12.05	C17[M+H]^+^	C_51_H_80_N_10_O_15_	
	1095.5682		C17[M+Na]^+^		
Fengycins					
Fengycins A					
F1	1435.7688	11.20	C14[M+H]^+^	C_70_H_106_N_12_O_20_	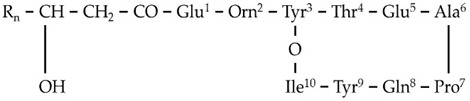
	718.3880		C14[M+H]^2+^		
F2	1449.7848	11.54	C15[M+H]^+^	C_71_H_108_N_12_O_20_	
	725.3958		C15[M+H]^2+^		
F3	1463.8005	12.02	C16[M+H]^+^	C_72_H_110_N_12_O_20_	
	732.4037		C16[M+H]^2+^		
F4	1477.8176	12.27	C17[M+H]^+^	C_73_H_112_N_12_O_20_	
	739.4125		C17[M+H]^2+^		
Fengycins B					
F5	1463.8009	11.54	C14[M+H]^+^	C_72_H_110_N_12_O_20_	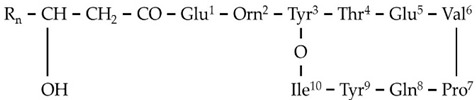
	732.4033		C14[M+H]^2+^		
F6	1477.8173	11.84	C15[M+H]^+^	C_73_H_112_N_12_O_20_	
	739.4122		C15[M+H]^2+^		
F7	1491.8318	12.32	C16[M+H]^+^	C_74_H_114_N_12_O_20_	
	746.4196		C16[M+H]^2+^		
F8	1505.8480	12.64	C17[M+H]^+^	C_75_H_116_N_12_O_20_	
	753.4275		C17[M+H]^2+^		
Gageotetrins					
Gageotetrins A					
G1	487.3378	13.95	C14[M+H]^+^	C_25_H_46_N_2_O_7_	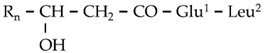
G2	501.3536	14.49	C15[M+H]^+^	C_26_H_48_N_2_O_7_	
Gageotetrins B					
G3	727.5231	16.14	C14[M+H]^+^	C_38_H_70_N_4_O_9_	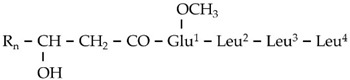
Gageotetrins C					
G4	699.4916	14.69	C13[M+H]^+^	C_36_H_66_N_4_O_9_	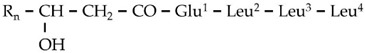
G5	713.5078	15.61	C14[M+H]^+^	C_37_H_68_N_4_O_9_	
Bacilotetrins					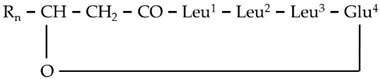
Bacilotetrin A					
Bt1	695.4964	17.01		C_37_H_66_N_4_O_8_	
Bacilotetrin B					
Bt2	709.5120	17.43		C_38_H_68_N_4_O_8_	

## Data Availability

Representative TIC (ESI+) chromatograms of the *Bacillus* sp. PTA13 lipopeptide extract and fractions in ”*.raw” format can be freely accessed from the repository of the Pesticide Metabolomics Group (https://www.aua.gr/pesticide-metabolomicsgroup/Resources/default.html) (accessed on 18 October 2021).

## Supplementary Materials

The following are available online at https://www.mdpi.com/article/10.3390/metabo11120833/s1, Figure S1: Confrontation bioassays for the bioactivity assessment of selected olive tree endophytic bacterial isolates to *Colletotrichum acutatum* species complex PLS 90 (wild type) and PLS 88 (resistant to PPPs) isolates, Figure S2: Agarose gel electrophoresis of PCR products of selected olive tree endophytic bacterial isolates following amplification with the universal primers 27F and 534R that amplify the positions 27 and 534 of the bacterial 16S rRNA genes, respectively, Figure S3: *Bacillus* sp. PTA13 growth rate and its lipopeptide-producing capacity in the time-course study, Figure S4: TIC (ESI+) chromatograms of the upper and lower phases of the biphasic solvent systems 12–17, Figure S5: TLC chromatogram displaying the separation between the fengycin and bacillomycin groups produced by the olive tree endophytic *Bacillus* sp. PTA13 isolate, Figure S6: TIC (ESI+) of the combined CPC fractions displaying the gradual elution of the olive tree endophytic *Bacillus* sp., Figure S7: Olive tree tissues that were used for the isolation of endophytic microorganisms, Table S1: Biphasic solvent systems that were assessed for the fractionation of the *Bacillus* sp. PTA13 total lipopeptide (LP) extract, File S1: MS^1^ and MS^2^ of annotated *Bacillus* sp. PTA13 lipopeptides. The fragmentation patterns were used for their annotation, Data Set S1: *Bacillus*-produced lipopeptides.
