# Supplementary material for: Discovering the Next-Generation Plant Protection Products: A Proof-of-Concept via the Isolation and Bioactivity Assessment of the Olive Tree Endophyte Bacillus sp. PTA13 Lipopeptides
Source: Metabolites. 2021 Dec 2;11(12):833. doi: 10.3390/metabo11120833 (PMC8705366; doi:10.3390/metabo11120833)
Supplement: Supplementary file 1 [file metabolites-11-00833-s001.zip › File S1.pdf]

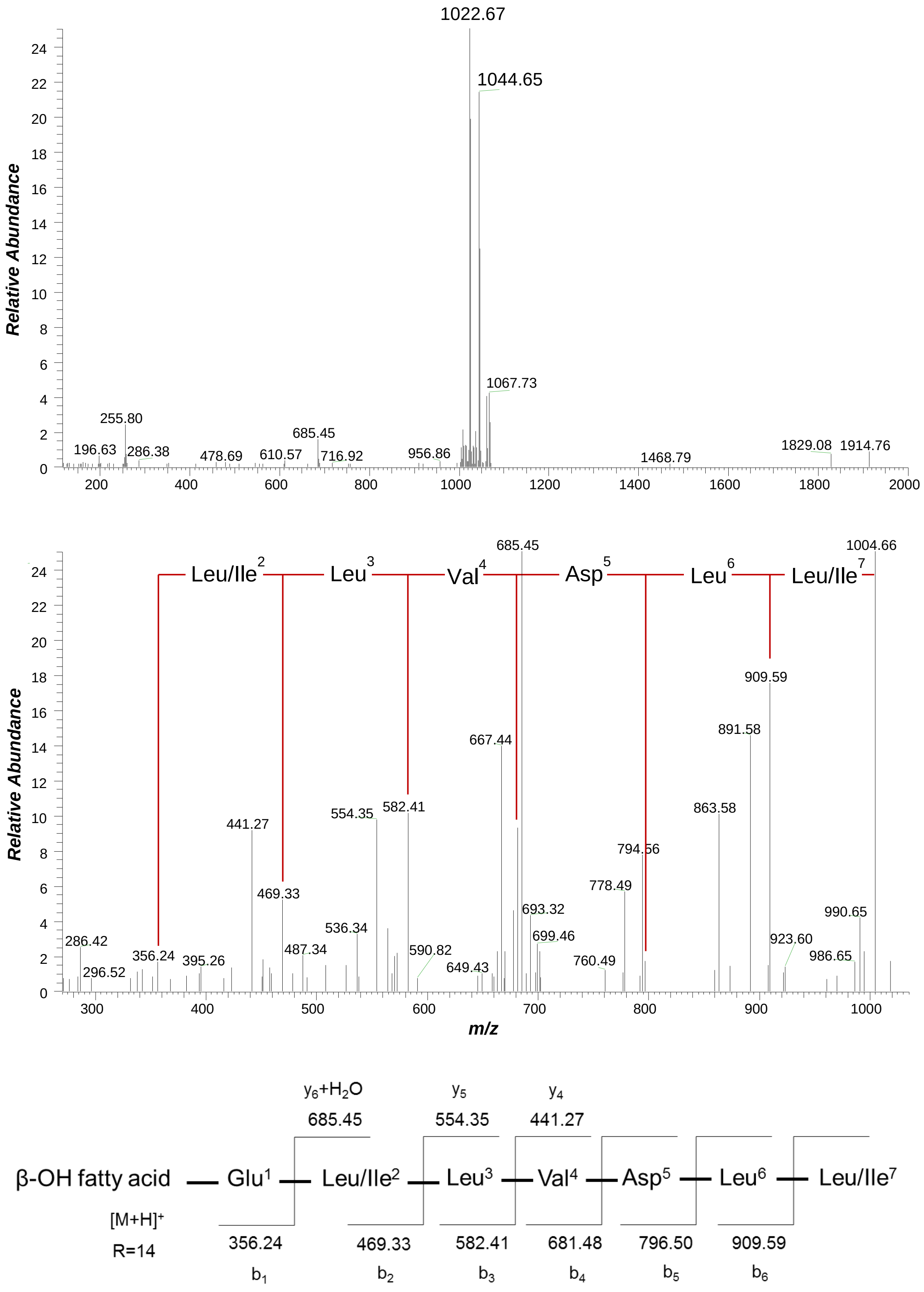

MS[1] (upper plot) and MS[2] (middle plot) of the annotated *Bacillus* sp. PTA13 lipopeptide (LP) surfactin C14 (S3, see Table 1). The fragmentation pattern was used for the identification of the LP (lower plot).

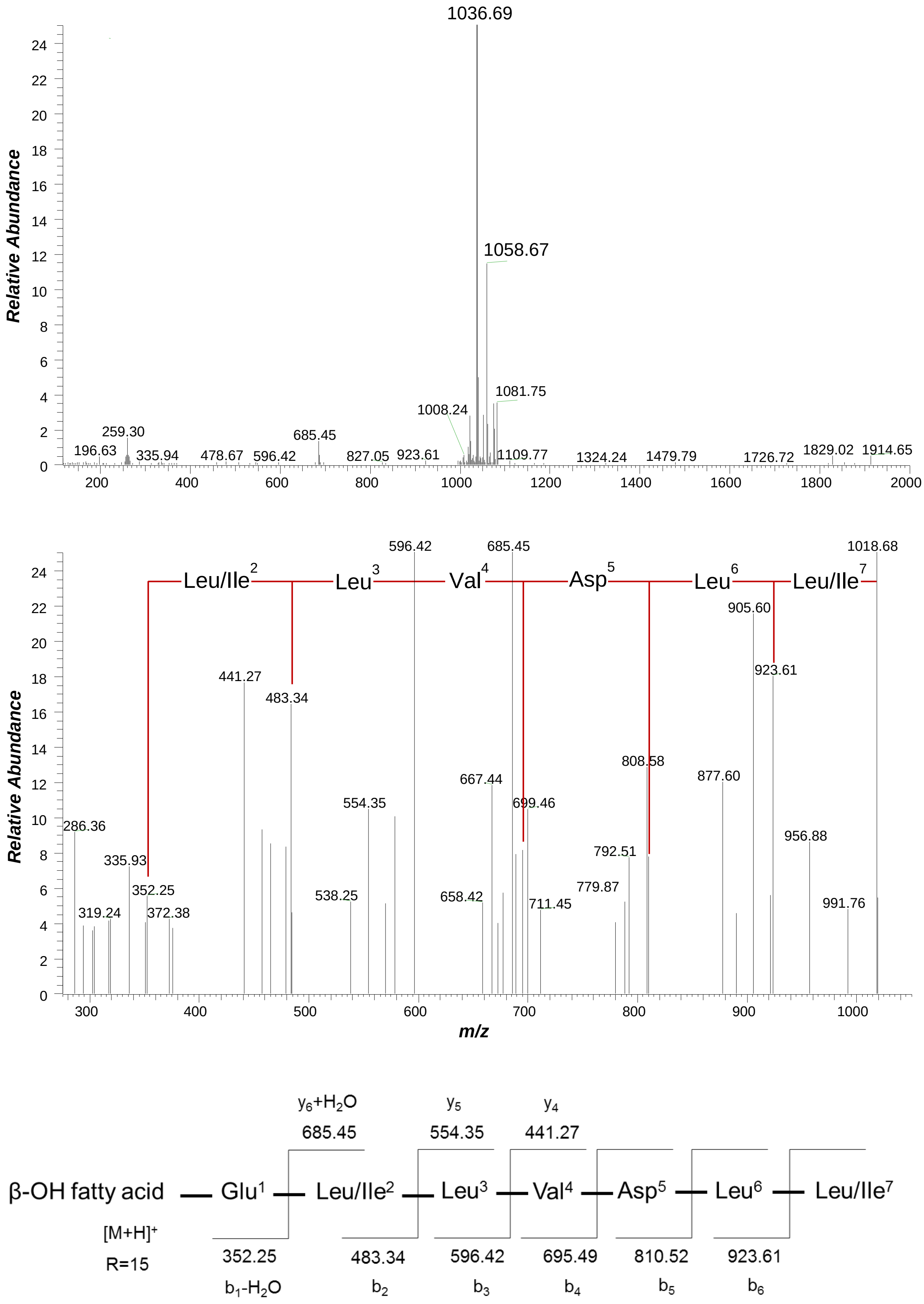

MS^1 (upper plot) and MS^2 (middle plot) of the annotated *Bacillus* sp. PTA13 lipopeptide (LP) surfactin C15 (S4, see Table 1). The fragmentation pattern was used for the identification of the LP (lower plot).

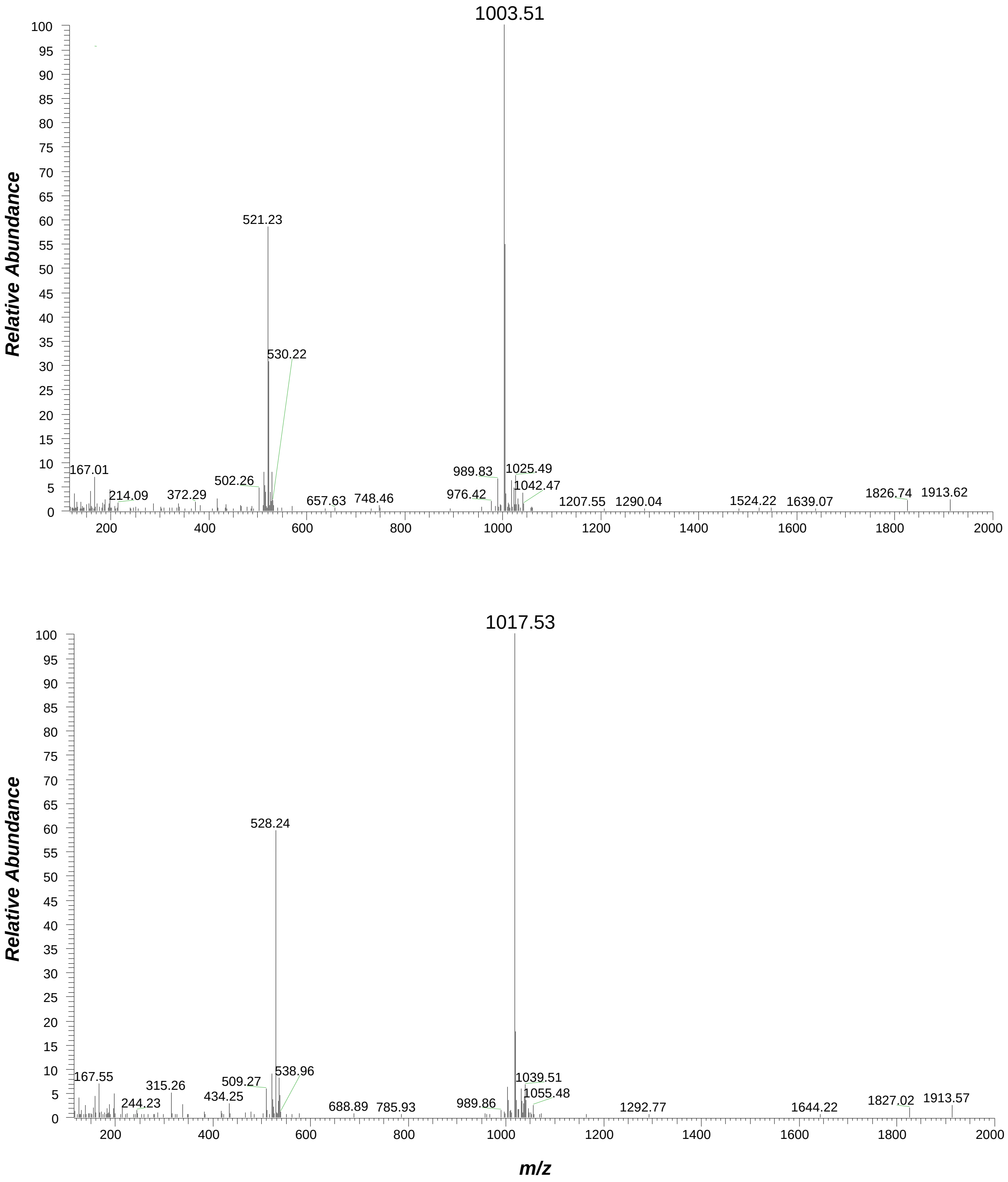

MS[1] of the annotated *Bacillus* sp. PTA13 lipopeptide (LP) bacillomycin D C12 (B1, see Table 1) (upper plot) and C13 (B2, see Table 1) (lower plot).

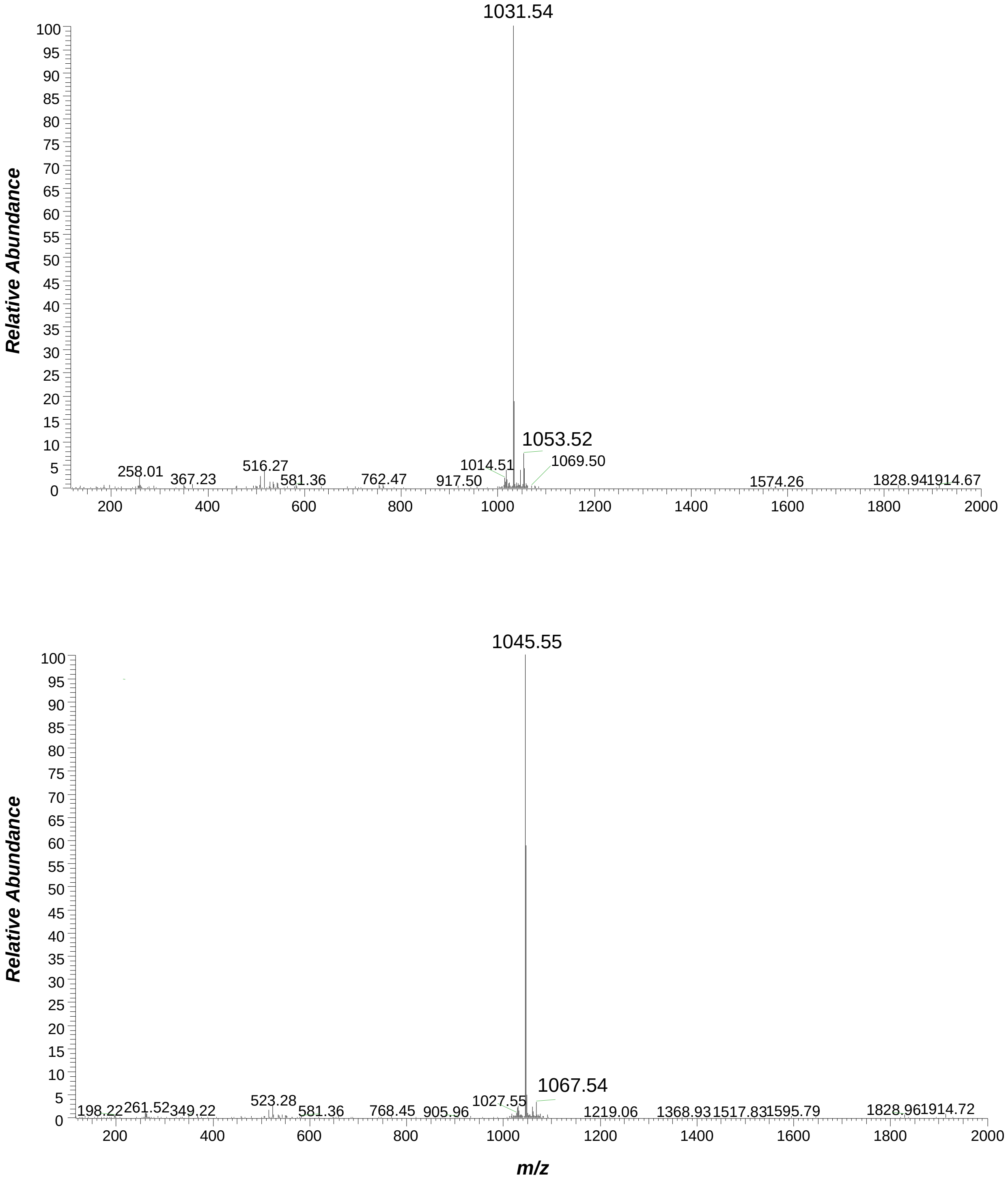

MS[1] of the annotated *Bacillus* sp. PTA13 lipopeptide (LP) bacillomycin D C14 (B3, see Table 1) (upper plot) and C15 (B4, see Table 1) (lower plot).

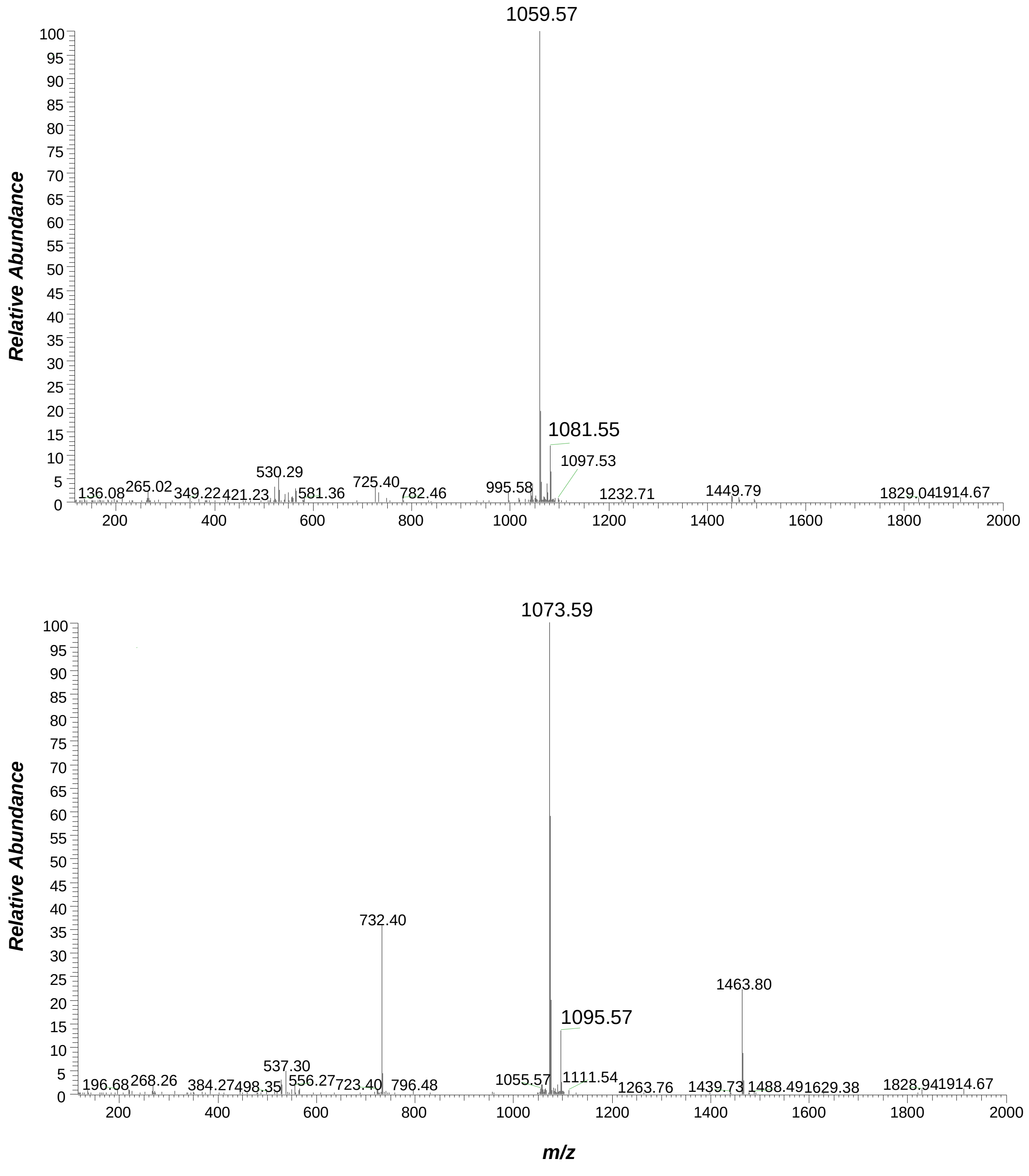

MS[1] of the annotated *Bacillus* sp. PTA13 lipopeptide (LP) bacillomycin D C16 (B5, see Table 1) (upper plot) and C17 (B6, see Table 1) (lower plot).

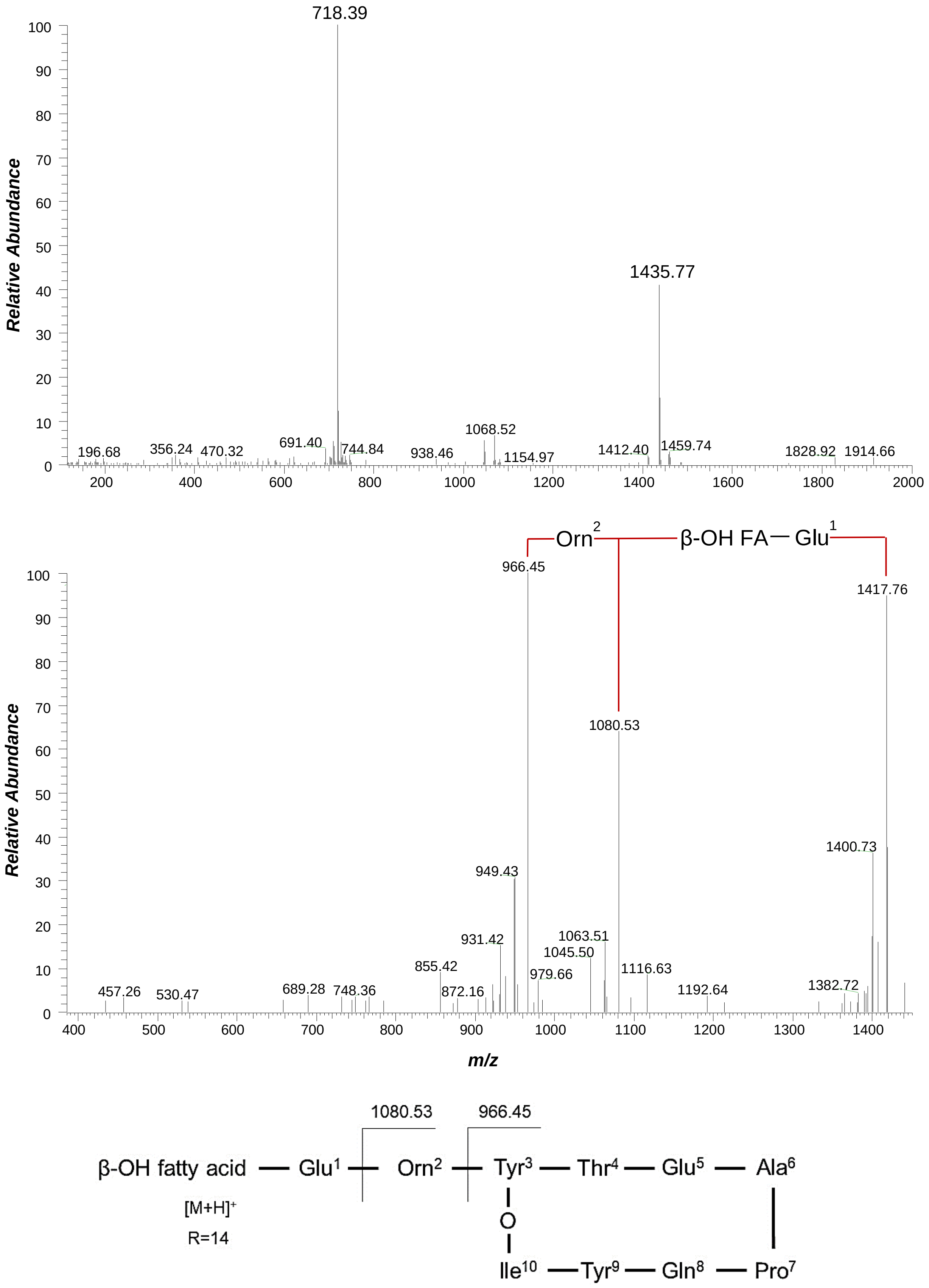

MS¹ (upper plot) and MS² (middle plot) of the annotated *Bacillus* sp. PTA13 lipopeptide (LP) fengycin A C14 (F1, see Table 1). The fragmentation pattern was used for the identification of the LP (lower plot).

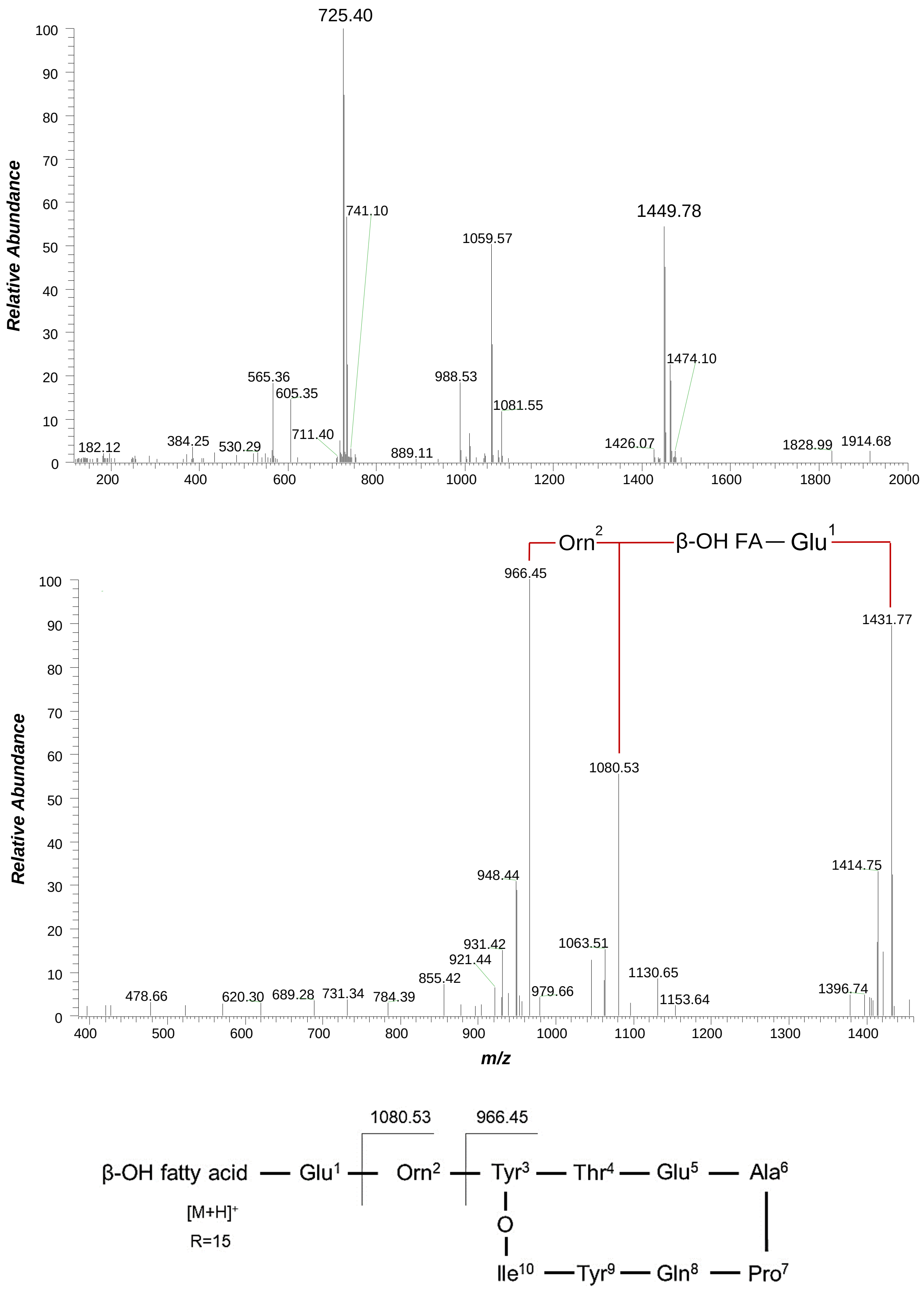

MS¹ (upper plot) and MS² (middle plot) of the annotated *Bacillus* sp. PTA13 lipopeptide (LP) fengycin A C15 (F2, see Table 1). The fragmentation pattern was used for the identification of the LP (lower plot).

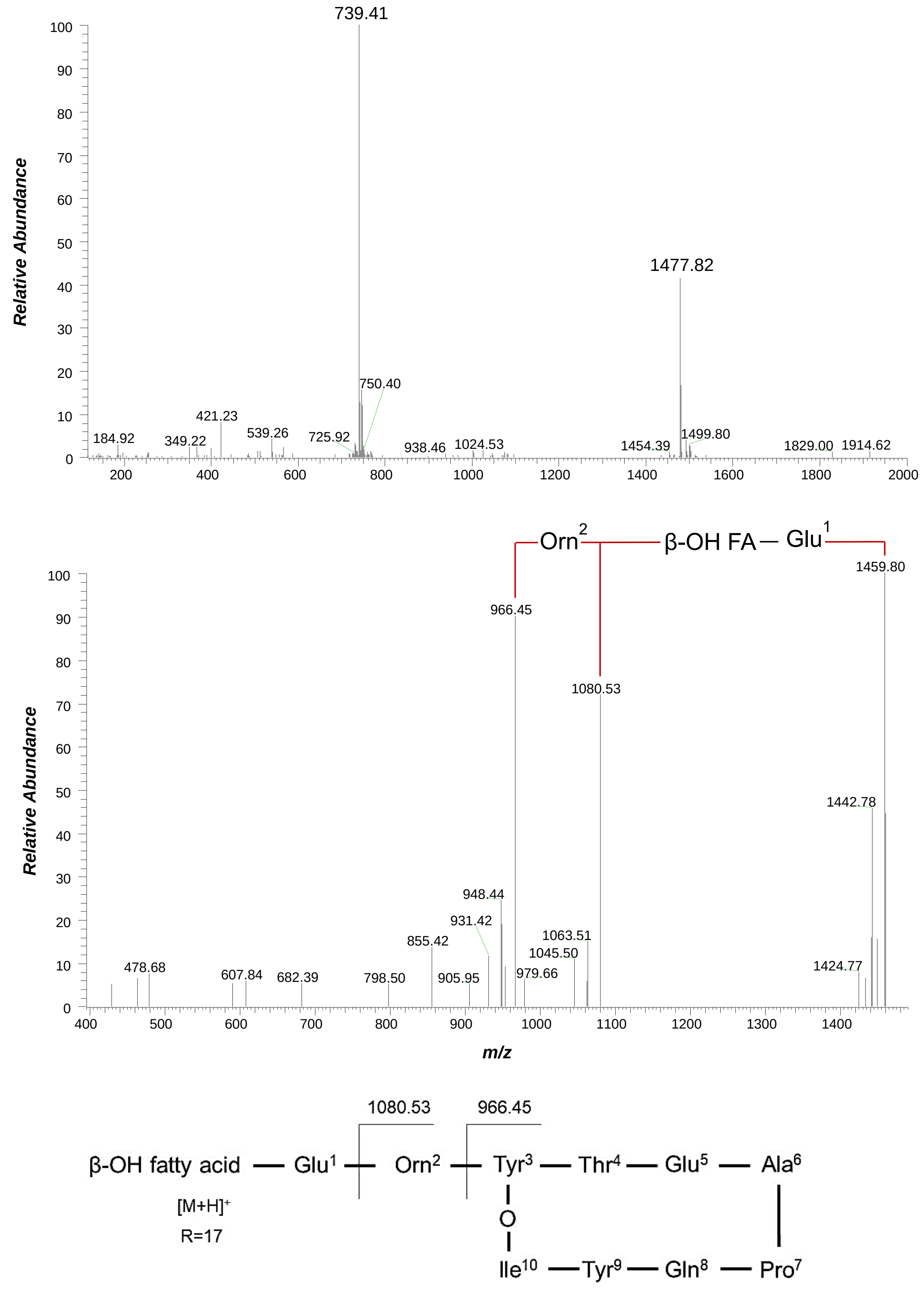

MS¹ (upper plot) and MS² (middle plot) of the annotated *Bacillus* sp. PTA13 lipopeptide (LP) fengycin A C17 (F4, see Table 1). The fragmentation pattern was used for the identification of the LP (lower plot).

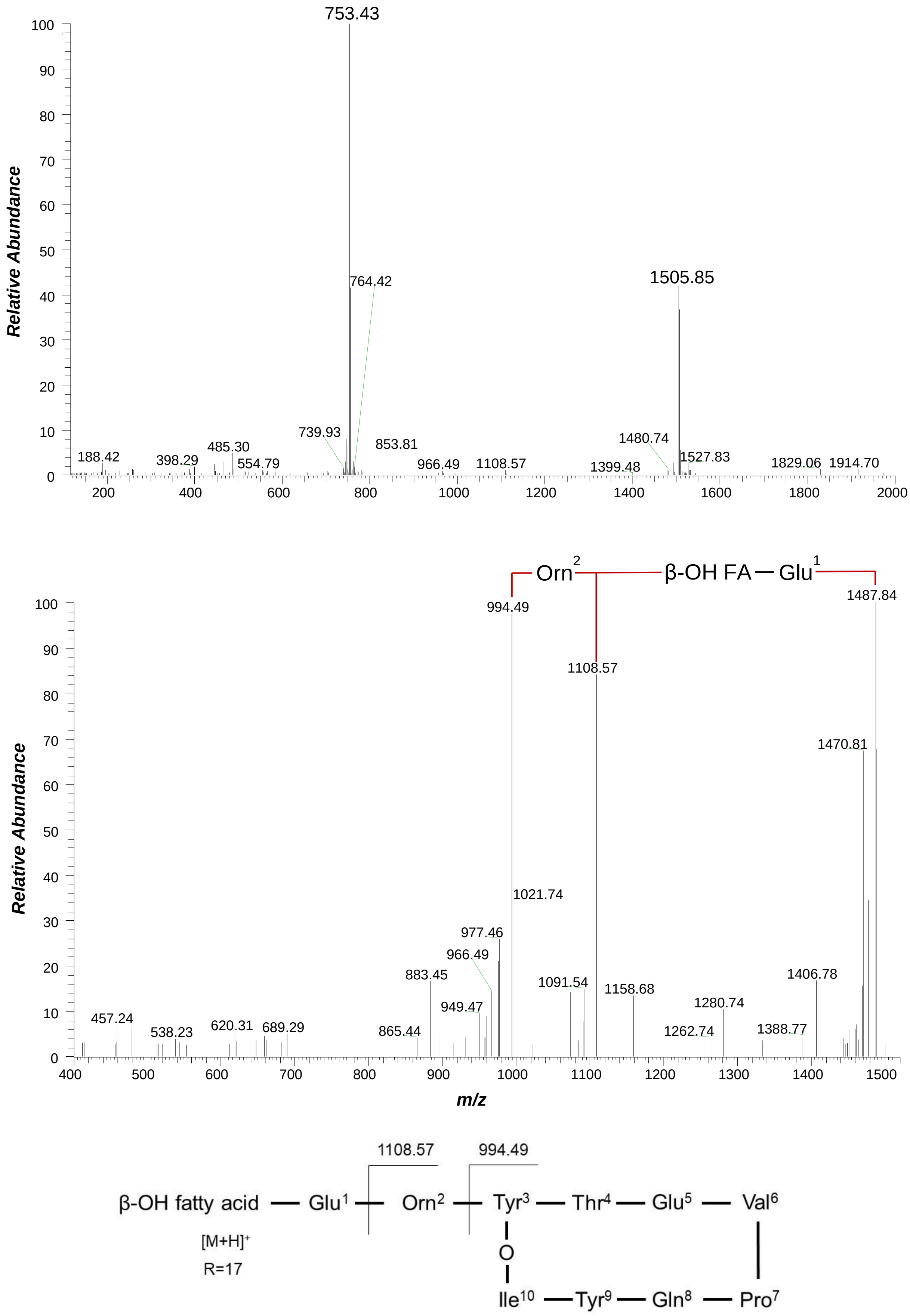

MS¹ (upper plot) and MS² (middle plot) of the annotated *Bacillus* sp. PTA13 lipopeptide (LP) fengycin B C17 (F8, see Table 1). The fragmentation pattern was used for the identification of the LP (lower plot).

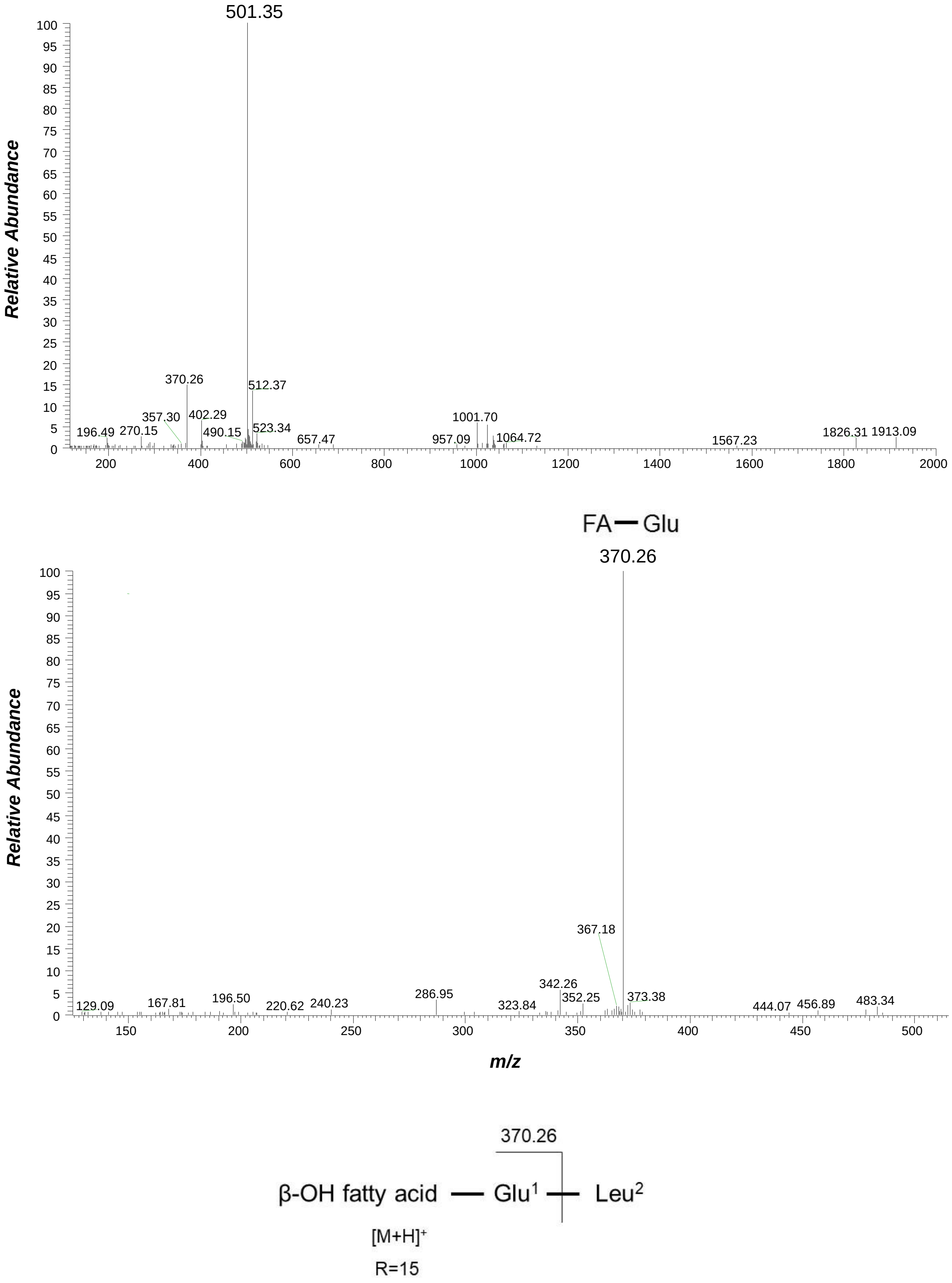

MS[1] (upper plot) and MS[2] (middle plot) of the annotated *Bacillus* sp. PTA13 lipopeptide (LP) gageotetrin A C15 (G2, see Table 1). The fragmentation pattern was used for the identification of the LP (lower plot).

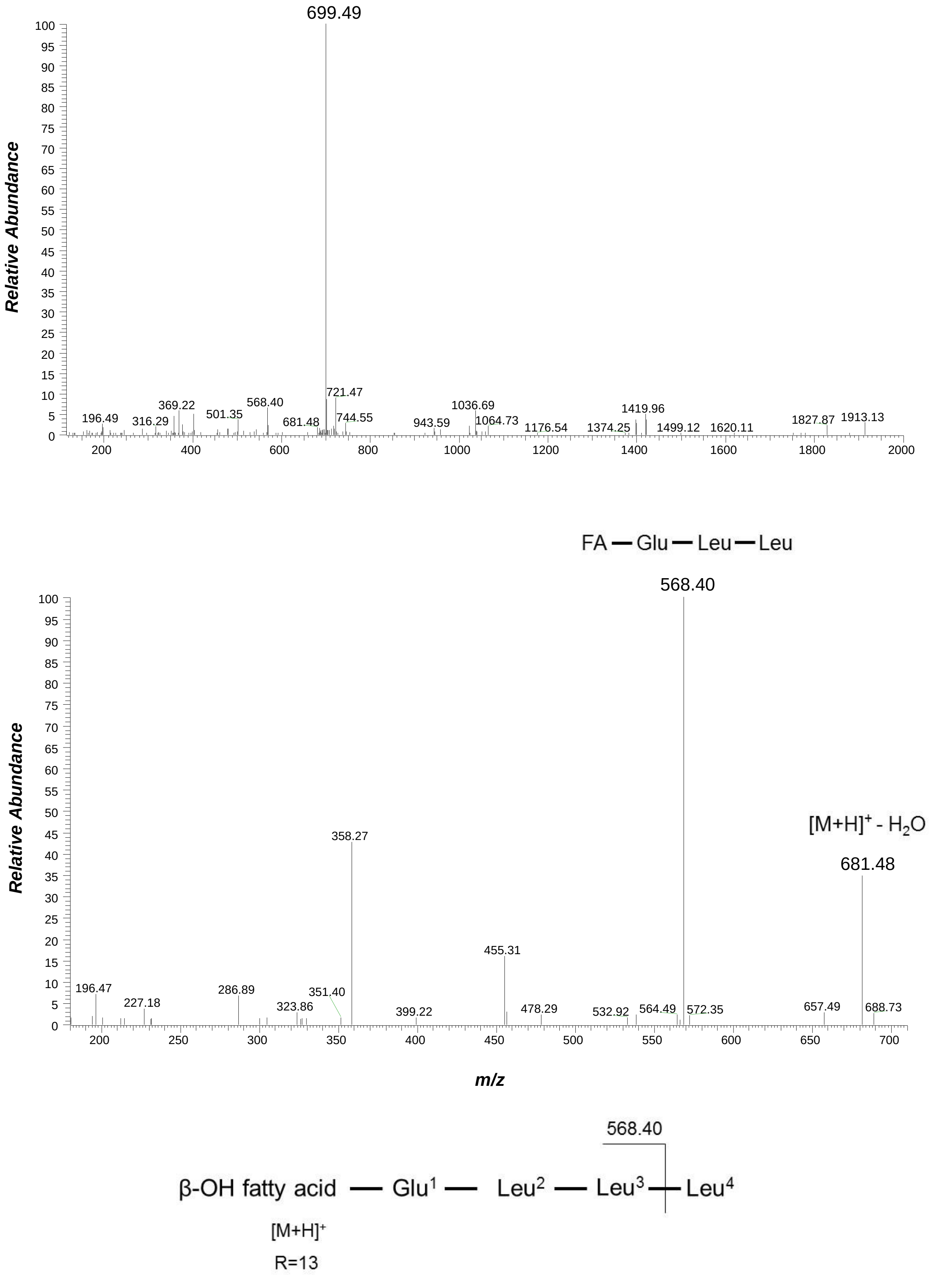

MS¹ (upper plot) and MS² (middle plot) of the annotated *Bacillus* sp. PTA13 lipopeptide (LP) gageotetrin C C13 (G4, see Table 1). The fragmentation pattern was used for the identification of the LP (lower plot).

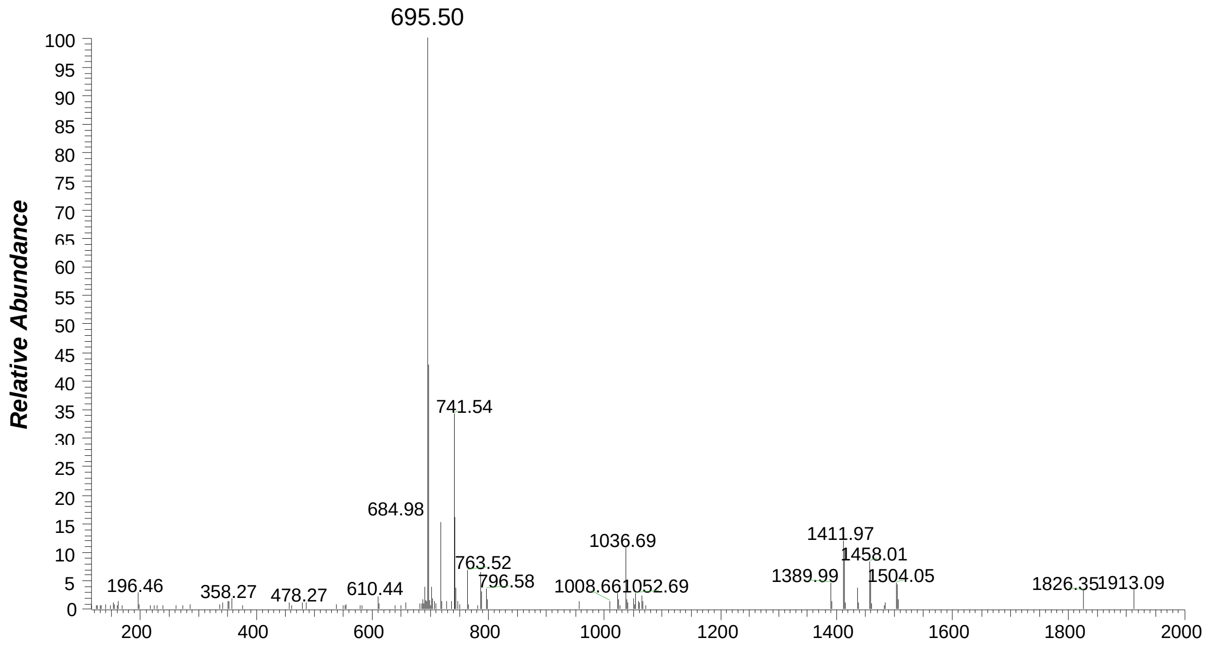

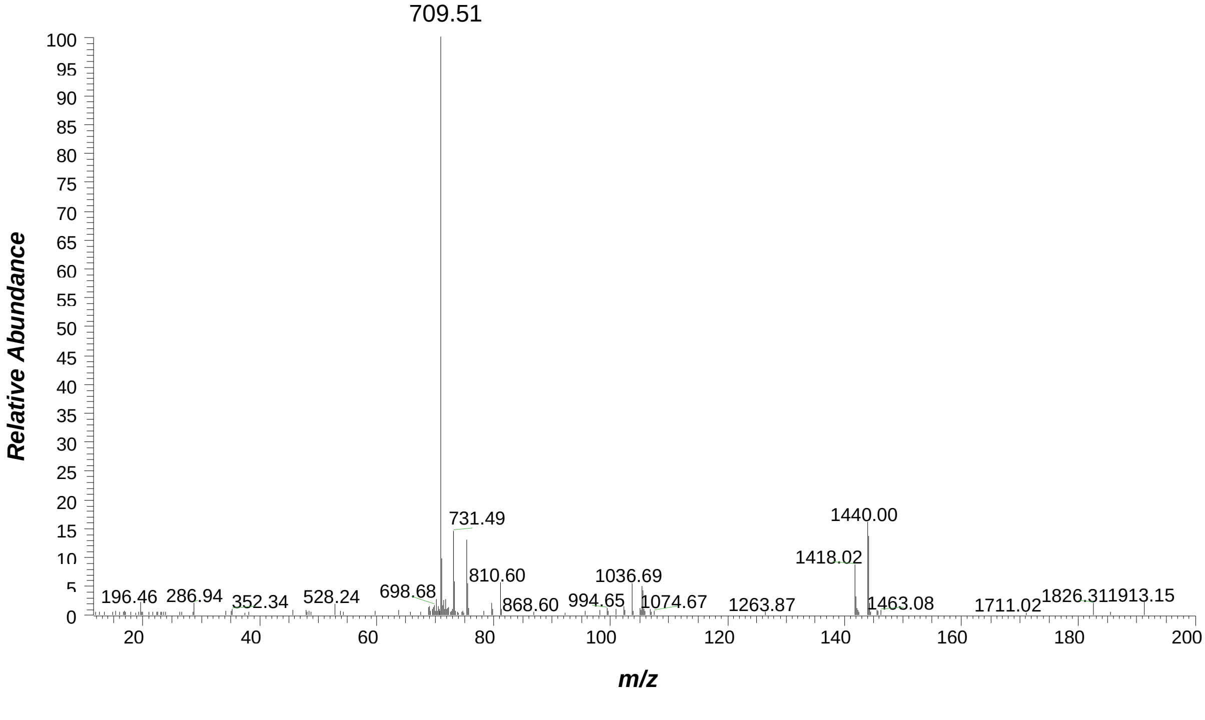

MS[1] of the annotated *Bacillus* sp. PTA13 lipopeptide (LP) bacilotetrin A (Bt1, see Table 1) (upper plot) and B (Bt2, see Table 1) (lower plot).
