# Supplementary material for: Discovering the Next-Generation Plant Protection Products: A Proof-of-Concept via the Isolation and Bioactivity Assessment of the Olive Tree Endophyte Bacillus sp. PTA13 Lipopeptides
Source: Metabolites. 2021 Dec 2;11(12):833. doi: 10.3390/metabo11120833 (PMC8705366; doi:10.3390/metabo11120833)
Supplement: Supplementary file 1 [file metabolites-11-00833-s001.zip › Supplementary Figures.pdf]

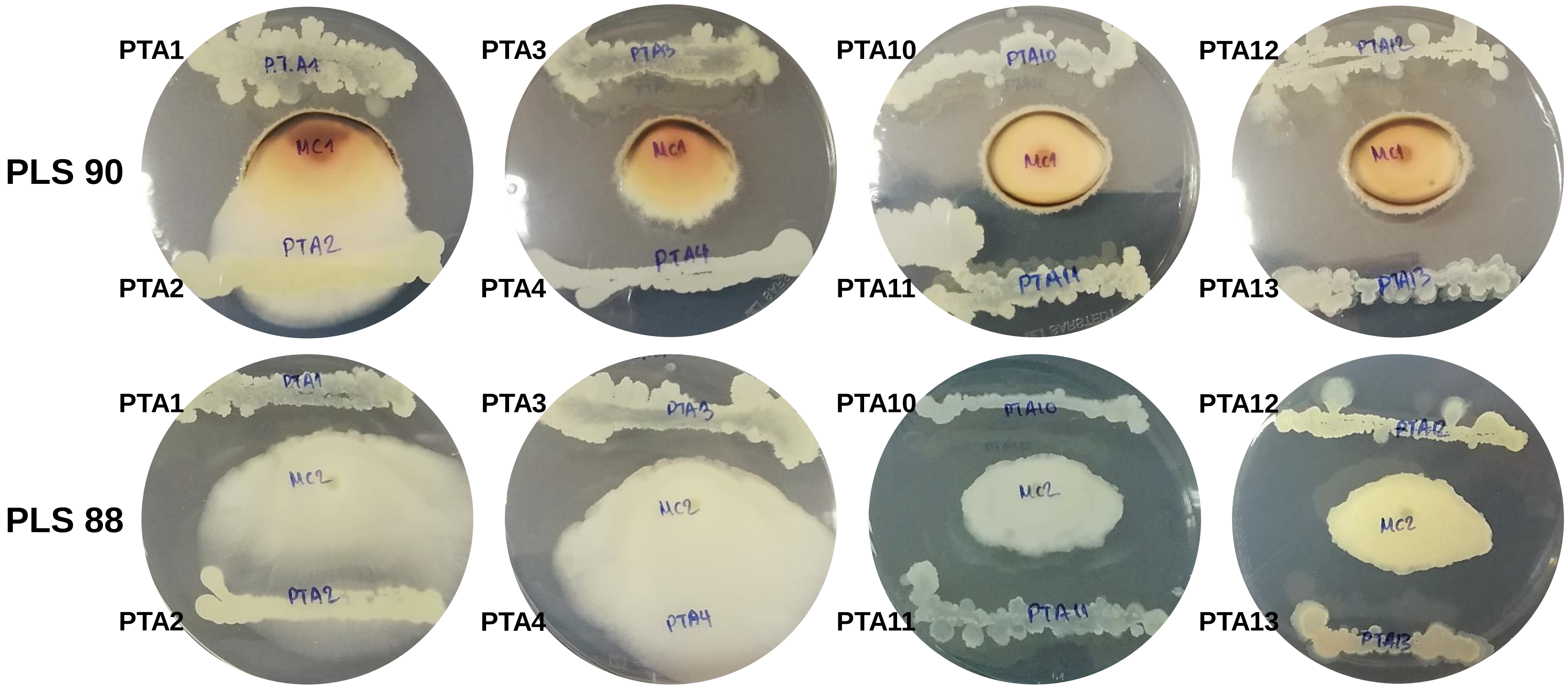

Figure S1. Confrontation bioassays for the bioactivity assessment of selected olive tree endophytic bacterial isolates to *Colletotrichum acutatum* species complex PLS_90 (wild type) and PLS_88 (resistant to PPPs) isolates. The formation of inhibition zones and the pattern of the fungal culture development confirmed the bioactivity of the isolates being tested with the exception of PTA2 and PTA4.

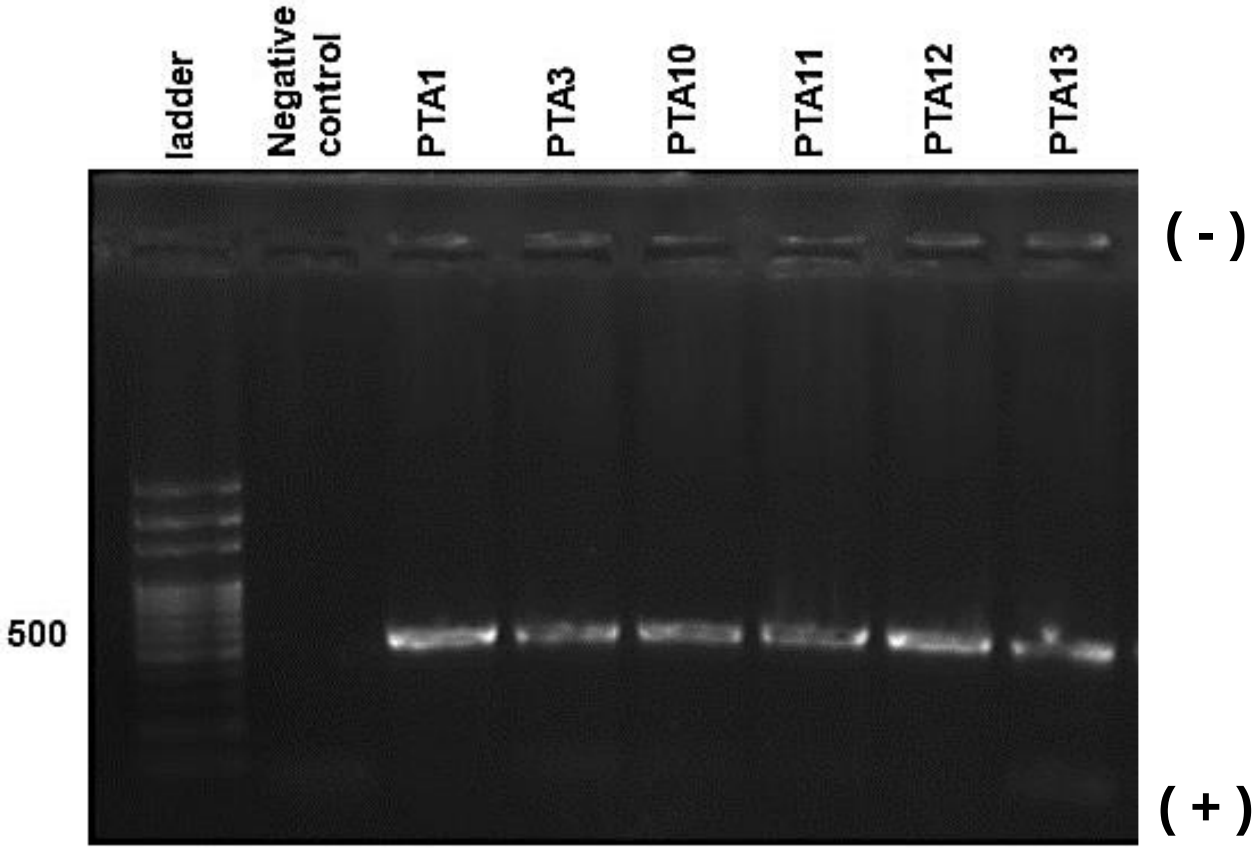

Figure S2. Agarose gel electrophoresis of PCR products of selected olive tree endophytic bacterial isolates following amplification with the universal primers 27F and 534R that amplify the positions 27 and 534 of the bacterial 16S rRNA genes, respectively. The bacteria were the most bioactive against *Colletotrichum acutatum* species complex PLS_90 (wild type) and PLS_88 (resistant to PPPs) isolates based on confrontation bioassays. Electrophoresis was performed on 1% agarose gel.

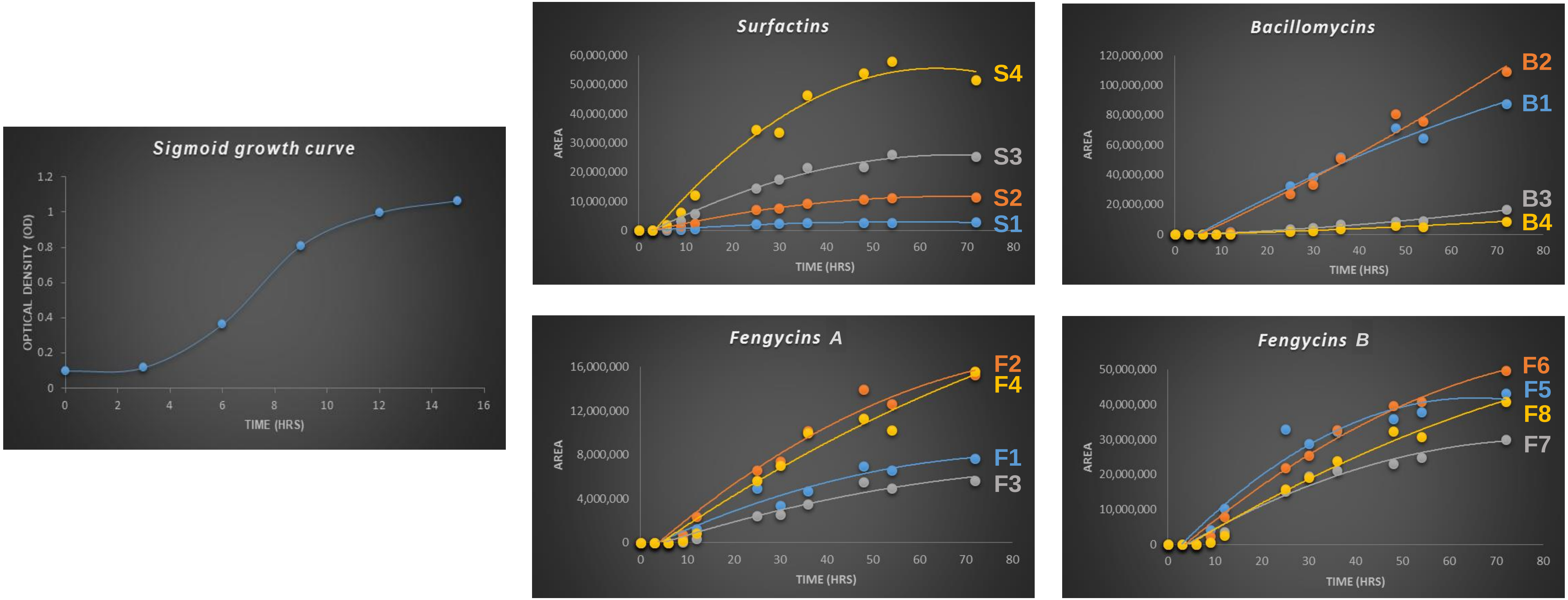

Figure S3. *Bacillus* sp. PTA13 growth rate and its lipopeptide-producing capacity in the time course. The production of the three major groups of *Bacillus* lipopeptides in the time course is displayed. An early onset of the surfactin biosynthesis is observed, whereas the onset of the biosynthesis of bacillomycins and fengycins is observed later on at 9 h post-inoculation.

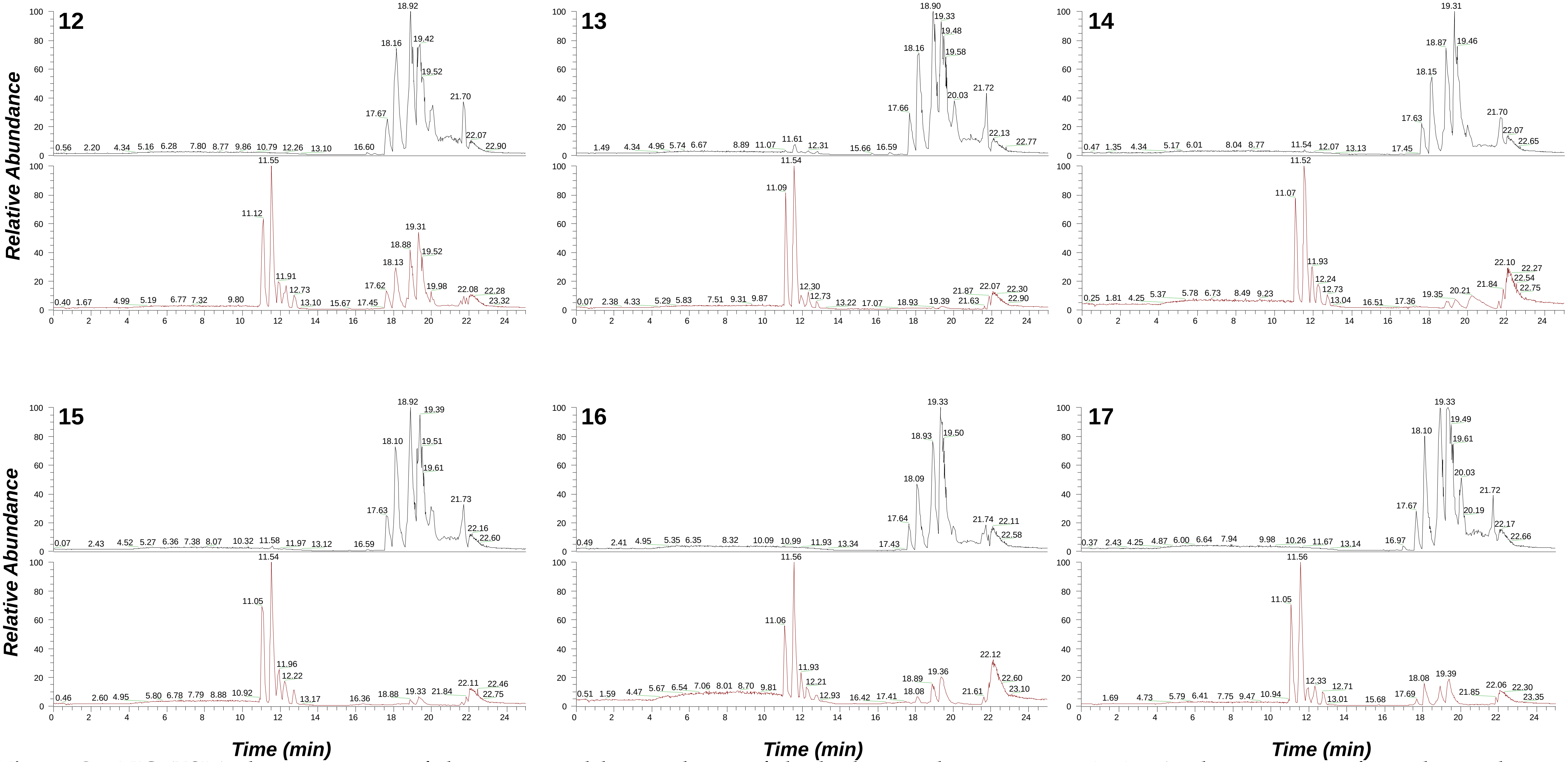

Figure S4. TIC (ESI+) chromatograms of the upper and lower phases of the biphasic solvent systems 12-17. Analyses were performed in order to evaluate the distribution of the components of the total lipopeptide extract into the two phases.

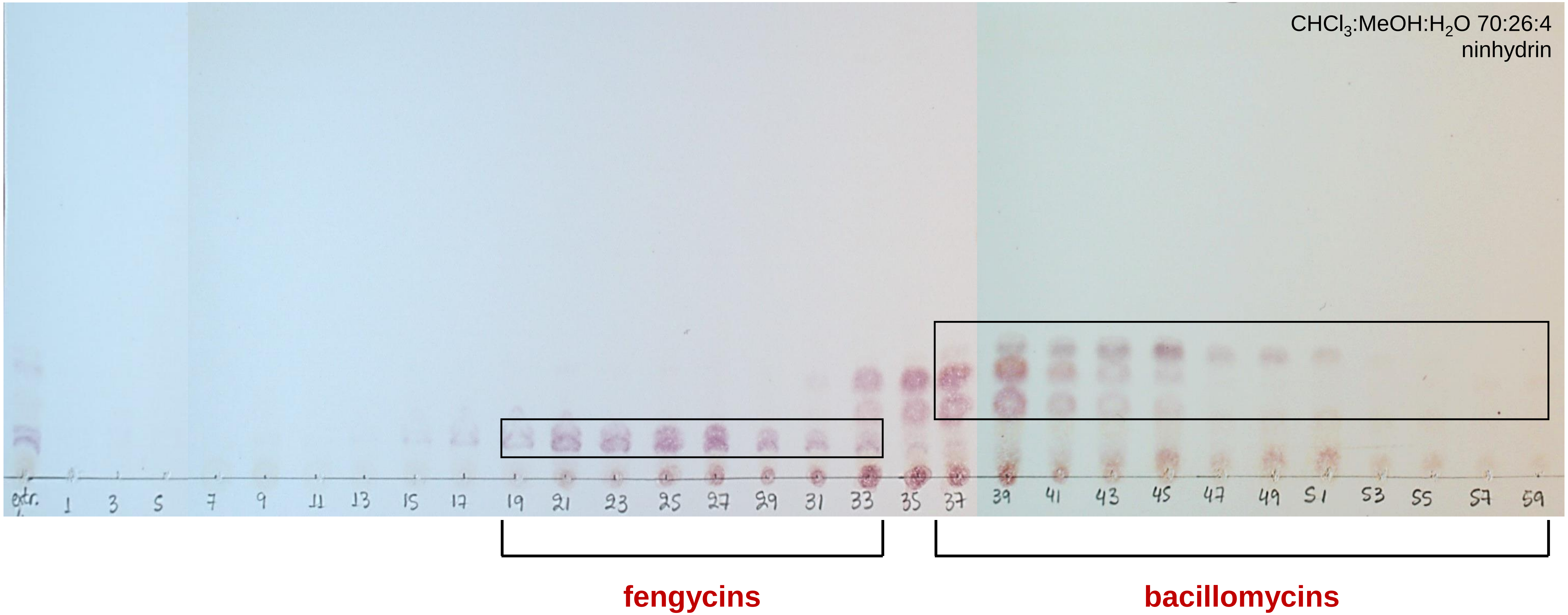

Figure S5. TLC chromatogram displaying the separation between the fengycin and bacillomycin groups produced by the olive tree endophytic *Bacillus* sp. PTA13 isolate. The fractions were collected applying size exclusion chromatography to the total LP extract's lower phase following liquid-liquid extraction. The TLC plate was sprayed with a ninhydrin solution, for the visualization of compounds containing primary amino groups, which react with ninhydrin to form purple-colored products.

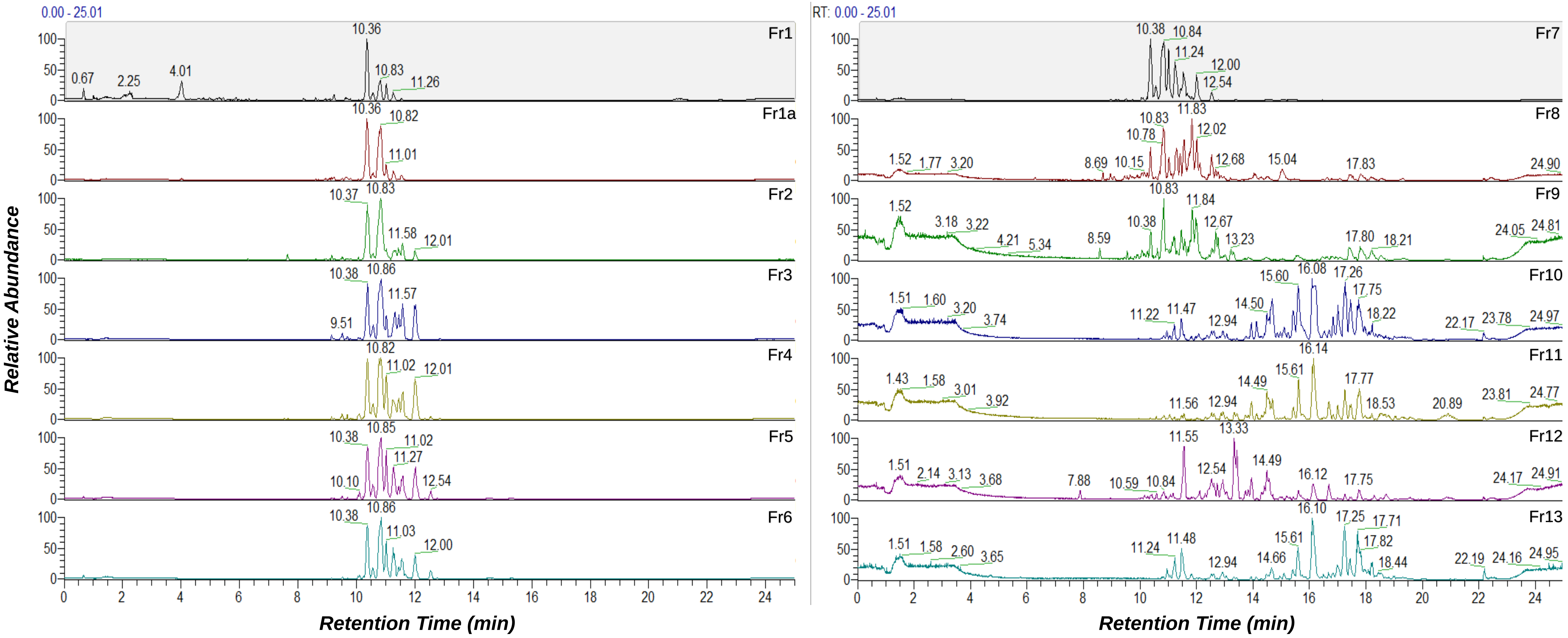

Figure S6. TIC (ESI+) of the combined CPC fractions displaying the gradual elution of the olive tree endophytic *Bacillus* sp. PTA13 lipopeptide (LP) groups of the total LP extract.

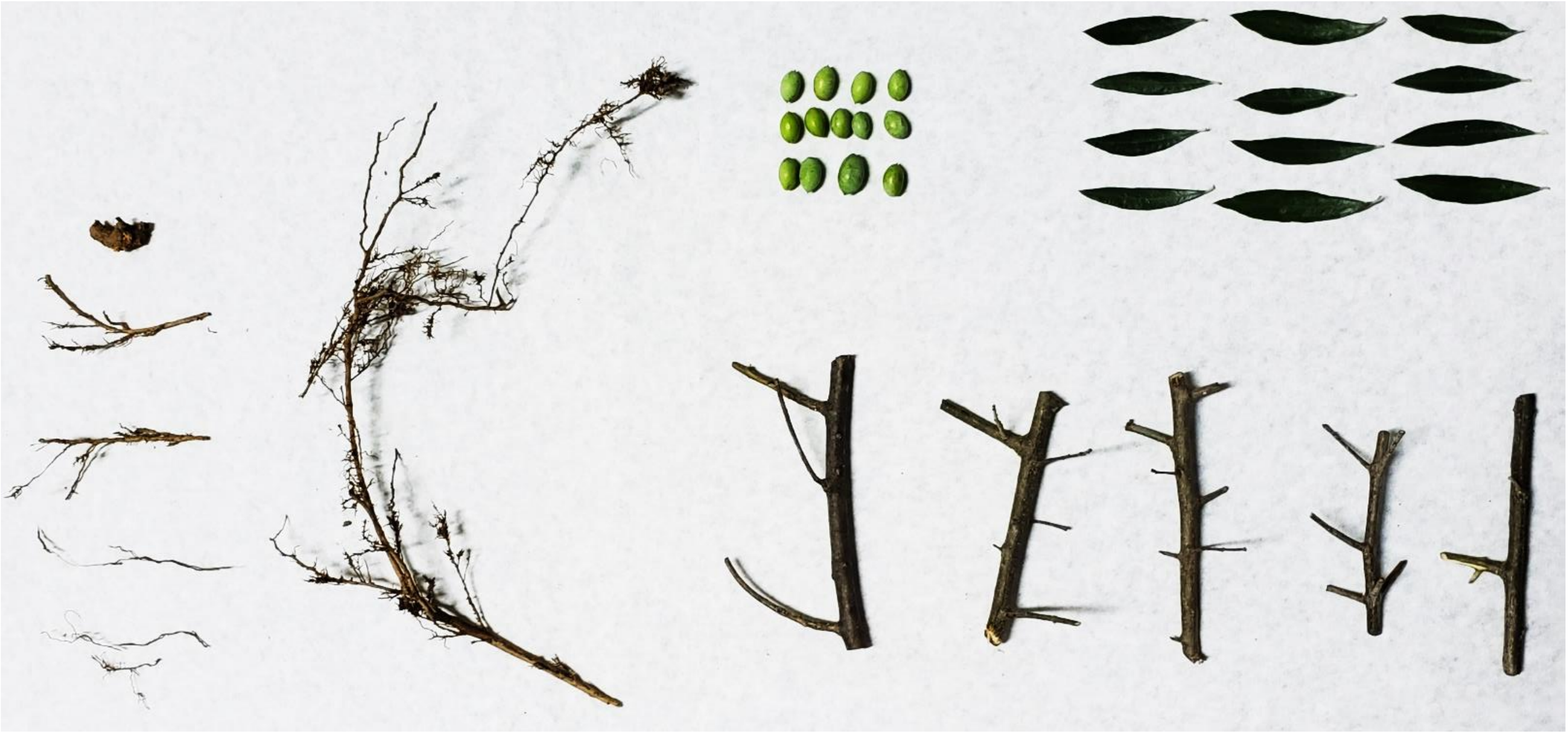

Figure S7. Olive tree tissues that were used for the isolation of endophytic microorganisms.
