# Supplementary material for: Discovering the Next-Generation Plant Protection Products: A Proof-of-Concept via the Isolation and Bioactivity Assessment of the Olive Tree Endophyte Bacillus sp. PTA13 Lipopeptides
Source: Metabolites. 2021 Dec 2;11(12):833. doi: 10.3390/metabo11120833 (PMC8705366; doi:10.3390/metabo11120833)
Supplement: Supplementary file 1 [file metabolites-11-00833-s001.zip › Table S1.pdf]

Table S1. Biphasic solvent systems that were assessed for the fractionation of the *Bacillus* sp. PTA13 total lipopeptide (LP) extract.

No.	Solvent System	Ratio
**1**	n-Hex/EtOAc/BuOH/EtOH/H_2O	1/11/3/5/10
**2**	n-Hex/EtOAc/BuOH/EtOH/H_2O	1/9/5/5/10
**3**	n-Hex/EtOAc/BuOH/EtOH/H_2O	1/7/7/5/10
**4**	n-Hex/EtOAc/BuOH/EtOH/H_2O	1/11/3/3/12
**5**	n-Hex/EtOAc/BuOH/EtOH/H_2O	1/9/5/3/12
**6**	n-Hex/EtOAc/BuOH/EtOH/H_2O	1/7/7/3/12
**7**	n-Hex/EtOAc/BuOH/EtOH/H_2O	2/8/0/4/6
**8**	n-Hex/EtOAc/BuOH/EtOH/H_2O	2/7/0/4/6
**9**	n-Hex/EtOAc/BuOH/EtOH/H_2O	2/6/2/4/6
**10**	n-Hept/EtOAc/MeOH/H_2O	0/1/0/1
**11**	n-Hept/EtOAc/MeOH/H_2O	1/0/1/0
**12**	n-Hept/EtOAc/MeOH/H_2O	1/1/1/1
**13**	n-Hept/EtOAc/MeOH/H_2O	1/3/1/3
**14**	n-Hept/EtOAc/MeOH/H_2O	1/4/2/3
**15**	n-Hept/EtOAc/MeOH/H_2O	2/3/2/3
**16**	n-Hept/EtOAc/MeOH/H_2O	3/2/2/3
**17**	n-Hept/EtOAc/MeOH/H_2O	4/1/2/3
